# A reference-grade wild soybean genome

**DOI:** 10.1038/s41467-019-09142-9

**Published:** 2019-03-14

**Authors:** Min Xie, Claire Yik-Lok Chung, Man-Wah Li, Fuk-Ling Wong, Xin Wang, Ailin Liu, Zhili Wang, Alden King-Yung Leung, Tin-Hang Wong, Suk-Wah Tong, Zhixia Xiao, Kejing Fan, Ming-Sin Ng, Xinpeng Qi, Linfeng Yang, Tianquan Deng, Lijuan He, Lu Chen, Aisi Fu, Qiong Ding, Junxian He, Gyuhwa Chung, Sachiko Isobe, Takanari Tanabata, Babu Valliyodan, Henry T. Nguyen, Steven B. Cannon, Christine H. Foyer, Ting-Fung Chan, Hon-Ming Lam

**Affiliations:** 1 Centre for Soybean Research of the State Key Laboratory of Agrobiotechnology and School of Life Sciences, The Chinese University of Hong Kong, Shatin, Hong Kong Special Administrative Region China; 2 BGI Genomics, BGI-Shenzhen, Shenzhen, 518083 Guangdong China; 3 Wuhan Institute of Biotechnology, Wuhan, 430075 Hubei China; 4 Department of Biotechnology, Chonnam National University, Gwangju, 550‐749 Jeonnam South Korea; 5 Kazusa DNA Research Institute, Kazusa-Kamatari, Kisarazu, 292-0818 Chiba Japan; 6 Division of Plant Sciences and National Center for Soybean Biotechnology, University of Missouri, Columbia, Missouri 65211 USA; 7 Corn Insects and Crop Genetics Research Unit, United States Department of Agriculture - Agricultural Research Service (USDA-ARS), Ames, Iowa 50011-4014 USA; 8 Faculty of Biological Sciences, Centre for Plant Sciences, University of Leeds, Leeds, LS2 9JT Yorkshire UK

## Abstract

Efficient crop improvement depends on the application of accurate genetic information contained in diverse germplasm resources. Here we report a reference-grade genome of wild soybean accession W05, with a final assembled genome size of 1013.2 Mb and a contig N50 of 3.3 Mb. The analytical power of the W05 genome is demonstrated by several examples. First, we identify an inversion at the locus determining seed coat color during domestication. Second, a translocation event between chromosomes 11 and 13 of some genotypes is shown to interfere with the assignment of QTLs. Third, we find a region containing copy number variations of the *Kunitz trypsin inhibitor* (*KTI*) genes. Such findings illustrate the power of this assembly in the analysis of large structural variations in soybean germplasm collections. The wild soybean genome assembly has wide applications in comparative genomic and evolutionary studies, as well as in crop breeding and improvement programs.

## Abstract (web summary)

Wild relatives of crop plants are invaluable germplasm for genetic improvement. Here, Xie *et al*. report a reference-grade wild soybean genome and show that it can be used to identify structural variation and refine quantitative trait loci.

## Introduction

Genomic information is the essential foundation of current crop improvement programs. Accurate information is required for effective tracking of genomic variations, mapping important quantitative trait loci (QTLs), and discovering novel alleles. These tasks are intrinsically dependent on the availability of a range of genetic resources and, most crucially, high-quality reference genomes^1^. Wild germplasm contributes a significant proportion of the genetic resources of major crop species^2,3^. Although reference genomes are currently available for two soybean cultivars, the Williams 82 (Wm82) genome has been most commonly used for a range of applications^4,5^. However, these approaches alone have limitations and cannot fully address questions regarding large structural variations or complex genomic rearrangements. A high-quality reference genome from wild soybean is also a crucial tool for use in such studies, because it increases the precision of population genetic analysis of complicated genomes. For example, genomic information is the essential foundation for understanding domestication-related events that involved wild germplasms^6–8^.

We have previously reported whole-genome sequencing data for wild and cultivated soybeans, and demonstrated the high genome diversity in wild soybean populations compared with cultivated soybean^9^. The genomic diversity of wild soybean has been confirmed and elaborated in reports by ourselves and others^6,7,10–13^. Despite several previous attempts at whole-genome assembly in wild soybeans^6–8^, a high-quality reference genome has remained elusive. In resolution of this important issue, we report here a high-quality genome for the wild soybean accession W05. W05 has previously been employed to identify several agronomically important QTLs, together with the identification of the causal gene conferring salt tolerance in wild soybean^8,14^. In this study, we not only demonstrate the power of W05 reference genome but also highlight its applicatibity in a wide range of comparative genomic and evolutionary studies, using a range of examples, such as the identification of large structural variations, QTLs, genes, and alleles. The advantages of combining high-quality reference genomes and optical mapping (OM) in studying structural variations among multiple accessions is also described.

## Results

### De novo sequencing and assembly

State-of-the-art whole-genome sequencing technologies were used to assemble a high-quality reference genome for wild soybean accession W05 with long contigs and high sequence fidelity. PacBio subreads (85.5 Gb) were error-corrected and de novo assembled into primary contigs (Supplementary Figure 1a). Sequences were then polished with PacBio subreads and Illumina paired-end reads (101.3 Gb) (Supplementary Figure 1b, Supplementary Table 1). The polished contigs are 989.7 Mb in length and are composed of 2281 sequences with a contig N50 (50% of the genome covered by contigs above this length) of 2.0 Mb. Details for assembly procedures can be found in Methods section.

To anchor polished contigs onto chromosomes with high accuracy, two complementary technologies: OM (Supplementary Table 2) and Hi-C sequencing (Supplementary Table 1) were employed. Based on the optical contigs generated with the nickases Nt.BspQI and Nb.BssSI (Supplementary Figure 1c), two-enzyme hybrid scaffolding was performed to generate OM-sequence hybrid scaffolds (hybrid scaffolds) (Supplementary Figure 1d). The hybrid scaffolds comprised 1438 sequences with a total length of 1019.8 Mb and a scaffold N50 of 13.9 Mb (Supplementary Figure 1d). In addition, Hi-C contact frequency derived from Hi-C sequencing was used to order and orient the polished contigs into Hi-C scaffolds (Supplementary Figure 1e). The resulting Hi-C scaffolds comprised 1161 sequences, with a total length of 989.8 Mb and a scaffold N50 of 48.5 Mb (Supplementary Figure 1e). Superscaffolds were generated by merging hybrid scaffolds and Hi-C scaffolds (Supplementary Figure 1f).

After gap filling and polishing, the final assembly for W05 is 1013.2 Mb in length, with 988.6 Mb unambiguous bases (Supplementary Figure 1g and Table 1). In total, 95.7% of sequences are anchored to 20 superscaffolds, corresponding to 20 chromosomes, whereas 43.6 Mb in 1098 contigs remain unplaced. The contig N50 of the final assembly is 3.3 Mb (Table 1). The longest contig of the W05 final assembly is 23.2 Mb in length, spanning 47.7% of chromosome 6. The contiguity of the W05 assembly is approximately a 17-fold improvement over the current reference genome Wm82_v2 and of similar quality as the recently published Chinese cultivated soybean reference genome of ZH13^5^ (Supplementary Table 3). Contig N50, scaffold N50, and total assembled genome size of other soybean genome assemblies^6,7,11,15^ were compared (Supplementary Table 3), but these genomes were not included in subsequent analysis, because they are highly fragmented.

**Table 1:** . Summary of W05 genome assembly and annotation

Categories	Type	Length (Mb)	No.	Percentage (%)
Assembly	Contigs	988.6	1870	−
	Contig N50	3.3	58	−
	Contig N90	0.4	432	−
	Scaffolds	1013.2	1118	−
	Scaffold N50	50.7	10	−
	Scaffold N90	38.4	19	−
Protein-coding genes	Total transcripts	−	89,477	100.0
	Function assigned transcripts	−	82,567	92.3
Non-coding RNAs	miRNA	0.036	288	0.004
	snRNA	0.216	1988	0.021
	rRNA	0.032	147	0.003
	tRNA	0.067	892	0.007
Transposable elements	Class I: Retroelements	359.6	−	35.5
	SINEs	1.1	−	0.1
	LINEs	13.3	−	1.3
	LTR elements	345.2	−	34.1
	Ty1/Copia	93.5	−	9.2
	Ty3/gypsy	248.0	−	24.5
	Others	3.8	−	0.4
	Class II: DNA transposons	74.8	−	7.4
	CMC-EnSpm	29.7	−	2.9
	MULE	27.9	−	2.8
	TcMar	0.8	−	0.1
	hAT	8.7	−	0.9
	Helitron	4.2	−	0.4
	Others	3.5	−	0.3
	Satellites	4.9	−	0.5
	Simple repeats	44.1	−	4.4
	Low complexity	3.1	−	0.3
	Unknown	59.8	−	5.9
	Total transposable elements	546.4	−	53.9

### Assembly evaluation

**Fig. 1:**
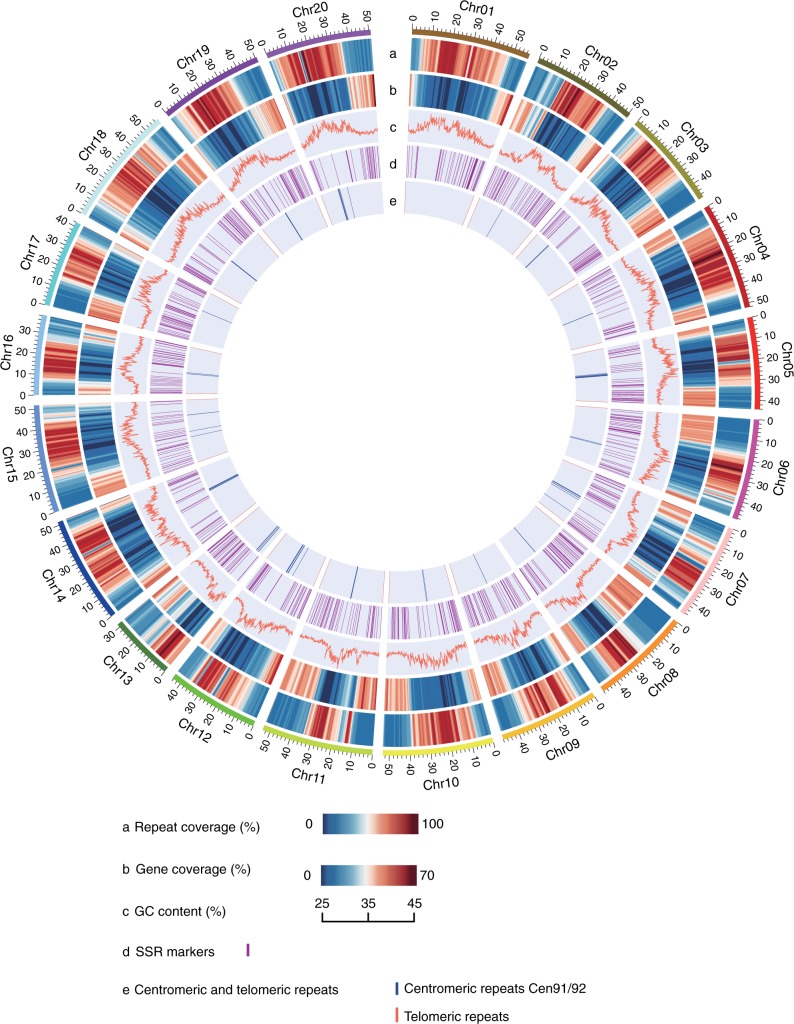
Distribution of W05 genomic features. The outer layer illustrates the 20 chromosomes of W05 in megabases (Mb). **a** Repeat coverage was calculated by the occupancy of repeat sequence in 1 Mb window (step size: 500 Kb). **b** Gene coverage was calculated by the occupancy of coding sequence in 1 Mb window (step size: 500 Kb). **c** GC content was calculated in a 200 Kb window. **d** Position of simple sequence repeat (SSR) markers were indicated in purple. Marker information could be found in Supplementary Data 1. **e** Presence of telomeric tandem arrays and cent91/92 soybean specific type centromeric repeats were marked in pink and blue, respectively

The completeness of this genome assembly was examined using the Benchmarking Universal Single-Copy Orthologs (BUSCO) evaluation score^16^. The completeness of W05 is comparable to the Wm82_v2 and ZH13 reference genomes (Supplementary Table 4). The mapping rate of the PacBio Isoform Sequencing (IsoSeq) full-length transcripts was 97.7% (Supplementary Table 5). Cent91/92 soybean-specific centromeric repeats^17^ were found in 19 chromosomes, except chromosome 1 (Fig. 1e). In addition, the *Arabidopsis*-type telomeric tandem repeat array^18^ (CCCTAAA/TTTAGGG repeats) was found at both distal ends for 13 chromosomes and at a single distal end for the remaining 7 chromosomes (Fig. 1e and Supplementary Table 6). In contrast, only 9 and 7 chromosomes of Wm82_v2 and ZH13, respectively, contain telomeric tandem repeats at both distal ends. The completeness of telomeres in the W05 genome is therefore improved compared with that of Wm82_v2 and ZH13 (Supplementary Table 6). GC content along each chromosome was calculated (Fig. 1c). Furthermore, using in silico PCR, 874 conserved, unique-site soybean simple sequence repeat markers were mapped to the W05 genome, which are evenly distributed on the 20 chromosomes (Fig. 1d and Supplementary Data 1).

### Genome annotation

Protein-coding genes and alternative spliced isoforms were annotated by combining evidence generated from RNA-seq/PacBio IsoSeq transcript mapping, homology-based protein mapping, and ab initio prediction. In total, 234.7 Gb of Illumina RNA-seq reads were collected from 31 samples at various development and physiological stages (Supplementary Data 2). PacBio IsoSeq libraries were constructed in order to generate 414,750 full-length and non-chimeric transcripts (Supplementary Table 7). In total, 89,477 protein-coding transcripts were annotated for 55,539 gene loci, with 69,455 transcripts (77.6%) having 5′-untranslated region (UTR) and 71,271 transcripts (79.7%) having 3′-UTR (Supplementary Table 8). In addition, 82,567 transcripts (92.3%) encode proteins that contain predicted functional domains (Table 1). Features of the annotated transcripts in W05 are similar to those of Wm82_v2 and ZH13^4,5^ (Fig. 1b and Supplementary Table 8). BUSCO evaluation shows that completeness of the W05 annotated gene set was comparable to that of Wm82_v2 and ZH13 (Supplementary Table 9). A total of 288 microRNAs (miRNA), 1988 small nuclear RNAs (snRNA), and 147 ribosomal RNAs (rRNA) were identified in the wild soybean genome (Table 1). In addition, 892 transfer RNAs (tRNA) were identified, representing anti-codons for all 20 types of amino acids.

### Identification and refinement of QTLs

One major application of the wild soybean reference genome is the identification of QTLs, genes, and alleles. We previously constructed a recombinant inbred (RI) population by crossing W05 to a cultivated soybean Union (C08)^8^, which shared the same recurrent parent with Wm82. To demonstrate that W05 can be used effectively as a reference genome for QTL mapping, we make use of our previous published phenotypic data together with new data of seed size and sequencing reads of 96 core RI lines^8^ to construct binmaps, and map QTLs using W05 genome or Wm82_v2 genome as reference. The relative genomic location and span of QTLs are comparable when either genome was used as the reference, with very few discrepancies (Supplementary Data 3). For example, the growth period QTL on chromosome 11 spans a 3.55 Mb region in the Wm82_v2 genome, but only 500 Kb in the W05 genome. This may be due to the low-quality assembly of Wm82_v2 in this region, as an ~3.8 Mb sequence within this region, which was originally present in Wm82_v1, was not anchored to the chromosome in Wm82_v2 (Supplementary Data 3).

The quality of the assembly down to the nucleotide level was assessed by examining known traits associated genes. For *Ncl*^8,19^, *Rj2/Rfg1*^20^, and *G*^21,22^ loci, the alleles in W05 match the observed phenotypes (Table 2, Supplementary Data 3 and 4). In addition, we have also identified known polymorphisms and additional alleles of the causal genes in the QTLs controlling growth period, flower color, seed coat color, and pubescence color in W05 (Table 2, Supplementary Data 3, 4).

**Table 2:** Predicted phenotypes based on genomic assemblies and observed phenotypes^*^

		W05			Wm82		
Trait	Locus	Allele type	Predicted phenotype	Observed phenotype	Allele type	Predicted phenotype	Observed phenotype
Salt tolerance	*Ncl*	Intact *GmCHX1*	Salt tolerant	Salt tolerant	TE-inserted *GmCHX1*	Salt sensitive	Salt sensitive
Nodulation	*Rj2*	*rj2(rfg1)*	Do not restrict neither *B. japonicum* nor *S. fredii*	Do not restrict neither *B. japonicum* nor *S. fredii*	*rj2(Rfg1)*	Restrict some strains of *S. fredii* but do not restrict *B. japonicum*	Restrict some strains of *S. fredii* but do not restrict *B. japonicum*
Flower color	*W1*	*W1*	Purple flower	Purple flower	*w1*	White flower	White flower
Seed coat color	*I*	*i*	Pigmented	Pigmented	*i* ^*i*^	Colorless	Colorless
Seed coat color	*G*	*G*	Stay green after seed maturation	Stay green after seed maturation	*g*	Do not stay green after seed maturation	Do not stay green after seed maturation

^*^Italicized text denoted gene loci, gene alleles, or species names

To investigate a more complex case, we examined the *I* locus on chromosome 8, which determines the pigmentation of the seed coat^23^, a major trait that was selected during domestication^21^. It was reported that the dominant allele in cultivated soybeans contains an inverted repeat of the chalcone synthase (*CHS*) gene cluster, which triggers posttranscriptional gene silencing (PTGS) and inhibits the expression of *CHS* gene family members in the seed coat; hence, resulting in colorless seed coat and yellow seeds^24^. Deletion that disrupts the inverted repeat *CHS* gene cluster in a revertant soybean accession resulted in seed coat color transition from colorless to pigmented^23^.

**Fig. 2:**
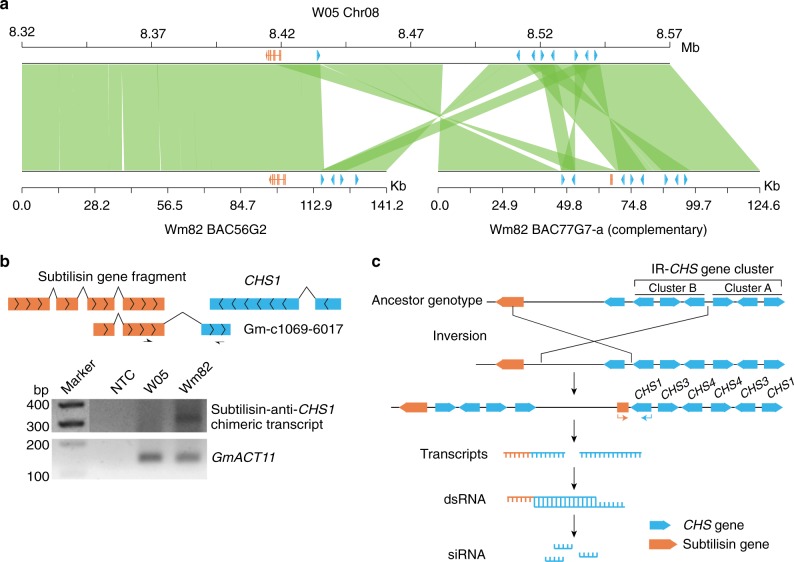
Causal structural variation that controls soybean seed coat pigmentation. **a** Sequence comparison between W05 genome and Wm82 bacterial artificial chromosome (BAC) sequences at the *I* locus region. *CHS* genes and subtilisin gene/gene fragments are indicated with blue and orange, respectively. **b** Top panel: cartoon shows the exon structure of the subtilisin gene fragment (orange), the *CHS1* gene (blue), and the Expressed Sequence Tag (EST) sequence Gm-c1069–6017. Positions of primers designed for PCR amplification of subtilisin-anti-*CHS1* chimeric transcript are indicated with black arrows. Bottom panel: PCR amplification of the subtilisin-anti-*CHS1* chimeric transcript. Experiment was repeated at least twice with independent samples. Marker: 1 Kb Plus DNA ladder (NEB, cat. N3200S). NTC, no template control. *GmACT11* is used as a housekeeping control. Unprocessed gel image is provided in Source Data file. **c** Proposed model for the generation of siRNAs originated from a large structural rearrangement in the *I* locus. *CHS* genes and the subtilisin gene/gene fragments are illustrated as blue and orange, respectively. Arrowheads indicated the direction of transcription that causes the formation of double-stranded RNA. Cluster A and B are named according to a previous report^23^. *IR-CHS* gene cluster: inverted repeat of *CHS* gene cluster

W05 has a pigmented seed coat, whereas C08 has a colorless seed coat. A seed coat color QTL that overlaps with the known *I* locus was mapped (Supplementary Data 3). The W05 reference genome possesses the same inverted repeat of the *CHS* gene cluster as Wm82 (Fig. 2a), indicating that the inverted repeat is not sufficient to explain the seed coat color change during domestication.

To identify the genetic variation that caused the seed coat color change during domestication, the W05 genome was compared with the two published bacterial artificial chromosomes of Wm82 that were previously used in *I* locus studies^23,25^ (the *I* locus region is poorly assembled in Wm82_v2), as well as the recently published ZH13 genome^5^. This comparative analysis reveals a complex structural rearrangement next to the *CHS* gene cluster in Wm82 and ZH13, which includes both inversion and gene duplication (Fig. 2a and Supplementary Figure 2). The inversion brought the promoter and the first four exons of a subtilisin gene (Glysoja.08G020214 in W05) to a position next to the *CHS* gene cluster in Wm82 and ZH13 (Fig. 2a and Supplementary Figure 2). This finding indicates that the subtilisin promoter may drive the expression of a chimeric transcript that reads through the subtilisin gene fragment and anti-*CHS1* gene region to cause PTGS of the *CHS* genes. This is in agreement with the previous speculation based on Expressed Sequence Tag data^26^. To validate this hypothesis, the subtilisin-anti-*CHS1* chimeric transcript was successfully amplified with PCR primers specific to both the subtilisin gene fragment and the anti-*CHS1* genes using complementary DNA from developing seed coat of Wm82 in a strand-specific manner (Fig. 2b). The subtilisin-anti-*CHS1* chimeric transcript was able to form double-stranded RNA with the sense *CHS* mRNAs, hence causing PTGS to inhibit the expression of *CHS* genes (Fig. 2c). Deletion of the *CHS* gene cluster B adjacent to the subtilisin gene fragment will disrupt PTGS and lead to the transition from a colorless seed coat to a pigmented seed coat, which is in agreement with the previous report^23^ (Fig. 2c).

### Major structural changes compared with cultivated genomes

Transposable elements (TEs) are repeated DNA sequences that make up of a significant proportion of plant genomes^27^. They are important source of variations for natural and artificial selection. Combining homology-based searches and de novo prediction, we identified 546.4 Mb of repeat elements (53.9% of the total assembled W05 genome) (Table 1, Fig. 1a). The most abundant type of TEs in the W05 genome is the long terminal repeat (LTR) retrotransposon element class, comprising 34.1% of the genome. The predominant LTR type is LTR/gypsy family.

Through whole-genome sequence comparisons of W05 with Wm82_v2 and ZH13, we have identified ~2300–3000 TE insertions for each accession (Supplementary Table 10). To identify Wm82- or ZH13-specific TE insertions, W05 was used as the reference genome. To identify W05-specific TE insertions, Wm82_v2 or ZH13 was used as the reference. In total, 361 and 350 W05 genes were found to contain TE insertions in Wm82_v2 and ZH13 genome, respectively. In contrast, 419 and 400 genes from Wm82_v2 and ZH13, respectively, were found to have TE insertions in W05 genome.

Gene Ontology (GO) enrichment analysis identified 29 and 8 enriched GO terms among the Wm82_v2 and ZH13 TE-affected genes, respectively. The enriched GO terms in both genomes mainly fall into the category of biological processes related to metabolism (Supplementary Data 5). Both sets contain GO:0044238 (primary metabolic process) and GO:0043170 (macromolecule metabolic process). For W05-specific TE-affected genes, there are three and nine GO terms enriched relative to ZH13 and Wm82, respectively. Unlike the two cultivated soybean references, the enriched GO terms mainly fall into the category of binding-related molecular functions (Supplementary Data 5) including GO:0036094 (small molecule binding), GO:0000166 (nucleotide binding), and GO:1901265 (nucleoside phosphate binding).

High-quality reference genomes also allow confident genome-wide detection of large structural variations, which cannot be achieved unambiguously solely by re-sequencing analysis. When comparing the genome sequence of W05 with Wm82_v2 and ZH13, good chromosome-to-chromosome collinearity relationships were found (Supplementary Figure 3). However, we also identified large structural variations ( >100 Kb inversions, intra-chromosomal translocations, and inter-chromosomal translocations) among the reference genomes (Supplementary Table 11). Compared with W05, there are 32 and 12 large structural variations in Wm82_v2 and ZH13, respectively (Supplementary Table 11). Nine of these variations are shared between Wm82_v2 and ZH13.

The largest structural variation in W05 relative to Wm82 and ZH13 is the inter-chromosomal reciprocal translocation in W05 between chromosomes 11 and 13. Previously, this translocation was detected in some *Glycine soja* accessions by fluorescent in situ hybridization (FISH)^28^. Analysis of the assembled genomes reveals this translocation event in W05 in comparison with Wm82 and ZH13 (Supplementary Figures 3 and 4). The structure of W05 chromosomes 11 and 13 is supported by Hi-C contact frequency matrix data, which shows higher intra-chromosomal contact frequency than inter-chromosomal contact frequency (Supplementary Figure 5). Consistent translocation breakpoints were identified when comparing W05 with ZH13 or Wm82_v1 but not with Wm82_v2 (Supplementary Figure 4). The discrepancy is most likely to be due to mis-assembly of Wm82_v2 in this region. Sequence comparisons revealed that the translocation breakpoint is located around 34.38 Mb on Chr11 and 27.06 Mb on Chr13 in the W05 genome (Supplementary Figure 4).

We also found that the QTLs controlling trailing growth, seed number per plant, and pod number per plant are located within the translocated regions (Supplementary Data 3). Therefore, the casual gene(s) are located on chromosome 13 in Wm82 and on chromosome 11 in W05 and some other soybeans (see below). If only Wm82 was used as the reference genome, these QTLs will be mis-placed to chromosome 13 in W05. Therefore, we have provided an important information for marker-assisted breeding or map-based cloning of the casual gene(s) for these QTLs.

### Reviewing large structural variations using OM

Although comparisons of the W05 and other reference genomes allows identification of many structural variations, these variations could be accession-specific. Therefore, we expanded our scope to analyze more soybean accessions from diverse origins, including cultivated soybeans popularized in China, the United States, and Japan, as well as wild soybeans originating from China and Korea (Supplementary Data 6). OM technology that can be used to study long-range genomic DNA up to 1 Mb in length was employed together with an in silico map based on our high-quality W05 genome to detect structural variations in diverse soybean accessions. We can thus demonstrate that the W05 reference genome enhances genome comparisons at the kilobase scale. As proof-of-concept, several loci exhibiting different structural features among soybean accessions were investigated, including inversion in the *I* locus, the translocation between chromosomes 11 and 13, a previously reported region specific to cultivated soybean on chromosome 15^7^, and gene copy number variation in a *Kunitz trypsin inhibitor* (*KTI*) gene cluster. Only soybean accessions with sufficient coverage and depth of optical contigs in the target regions were employed for comparative analysis (Supplementary Data 6).

**Fig. 3:**
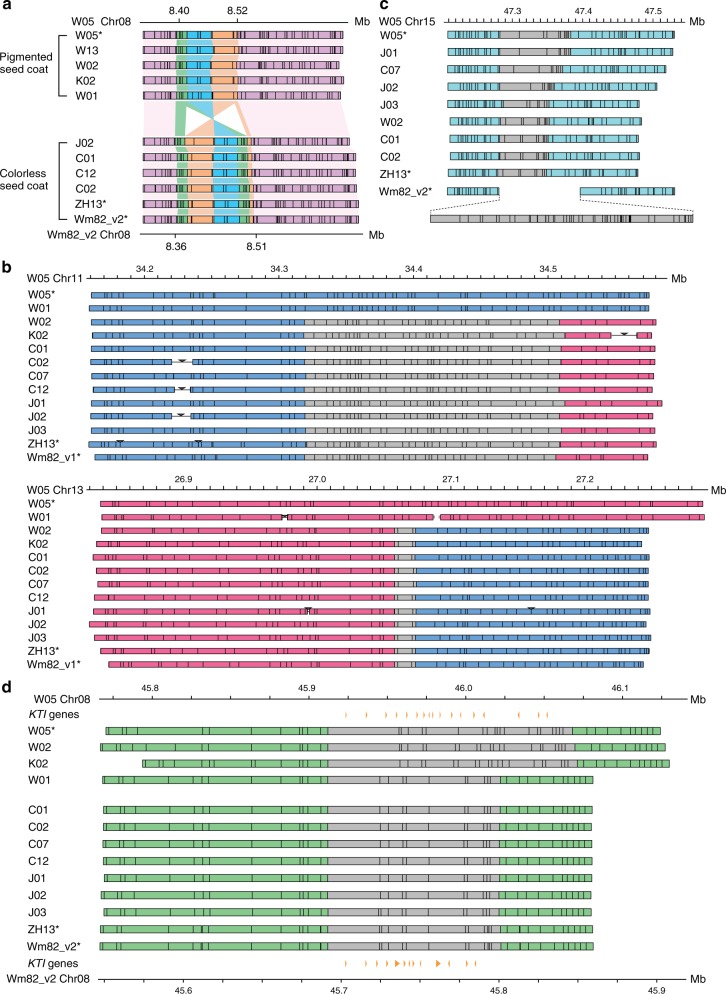
Large structural variations in soybean genomes detected by OM. **a** Seed coat pigmentation causal inversion in the *I* locus. Pink regions are the aligned flanking regions of the *I* locus. Aligned blocks in the *I* locus are painted in different colors to illustrate the inversion and duplication in accessions with colorless seed coat. **b** Reciprocal inter-chromosomal translocation between chromosomes 11 and 13. Segments in blue and red are regions homologous to the W05 chromosomes 11 and 13, respectively. Segments in gray contains optical signals that cannot be aligned to the W05 in silico map. **c** A previously reported cultivated soybean-specific region on chromosome 15^7^. Blue regions are the aligned flanking region of the previously proposed cultivated soybean-specific region. Segments that cannot be aligned with W05 in silico map are shown in gray. **d** Length polymorphism of a *KTI* gene cluster in chromosome 8. Orange triangles indicate the location of *KTI* genes in W05 (top track) and Wm82_v2 (bottom track), respectively. *KTI*, Kunitz trypsin inhibitor genes. Asterisks (*) next to the accession IDs indicate the use of in silico map instead of optical contigs

For the *I* locus, comparative analysis revealed that soybean accessions exhibiting a pigmented seed coat have no inversion (similar to W05), whereas those with colorless seed coats share the same inversion as Wm82 and ZH13 (Fig. 3a). To further confirm the OM results, the inversion junction that creates the subtilisin-anti-*CHS1* chimeric transcript was amplified from genomic DNA of different soybean accessions by PCR. Consistent with the OM results, inversion junction could be amplified from all soybean accessions with colorless seed coats but not from those with pigmented seed coats (Supplementary Figure 6).

Using Wm82_v1, ZH13, and W05 as references, the translocation event between chromosomes 11 and 13 were also detected. One (W01) out of three (W02, K02) wild soybean accessions share the same chromosome topology with W05. In contrast, all cultivated soybean accessions shared the same topology with the Wm82_v1 and ZH13 in silico maps, regardless of their origin whether they were popularized in China, the United States, or Japan (Fig. 3b). Furthermore, the regions at the junction of the translocation are reasonably well conserved among different accessions, which suggests that the translocation was a single evolutionary event.

Previously, a region specific to cultivated soybean (Gm15 46.0–46.5 Mb) was identified in a pan-genome study by mapping short reads of seven wild soybeans onto the Wm82_v1 genome^7^. We revisited this region and compared the optical contigs of different soybean accessions. Interestingly, the targeted region appears to have diversified among the different cultivated accessions that we have tested from China, the United States, and Japan, whereas only Wm82 possesses such a large insertion (Fig. 3c). This previously reported *Glycine max*-specific region was confirmed as Wm82 specific.

Comparisons of multiple reference genomes, together with the OM data, allow identification of gene copy number variations. KTI are anti-nutritional factors and are hence not desirable traits for selection in breeding purposes. Marker-assisted breeding has been used to introduce a null allele of *KTI* from an exotic soybean germplasm into two commercialized lines^29^. A *KTI* gene cluster was identified on chromosome 8 of W05 that contains 17 *KTI* genes. The optical signaling patterns in this region are similar for cultivated soybean accessions and diversify for wild soybean accessions. Wild soybean accessions W05, W02, and K01 possess long fragments, whereas W01 and all of the cultivated accessions tested (including Wm82) exhibited reduced size in this genomic region (Fig. 3d). In the annotated Wm82_v2 and ZH13 reference genomes, there are only 13 and 11 *KTI* genes, respectively, in this region (Supplementary Figure 7), implying that copy number variations of *KTI* genes might have occurred through artificial selection.

## Discussion

The availability of multiple high-quality reference genomes from diverse genetic backgrounds is a prerequisite for effective mining of crop genomes^30^, especially in studies involving wild germplasm. The current Wm82_v2 reference genome alone cannot provide conclusive answers to questions regarding large structural variations and complex genomic rearrangement. A new version of Wm82 genome is under construction (www.soybase.org), which will improve the quality over the current version. However, comparative genomic analysis using only the reference genome of Wm82 may still fail to uncover wild-specific genetic variations, as these may have been lost during domestication or artificial selection^7^. Here we report the assembly of a high-quality reference genome of a wild soybean accession W05, which has many distinguishing features compared with the two existing reference genomes of cultivated soybeans (see Results).

Wild soybeans exhibit agronomic traits that are different to cultivated soybean (e.g., smaller seed size, higher pod number, and seed number per plants, etc.). We have identified QTLs related to key yield components. A seed size QTL was reported and the proper chromosomal locations of the QTLs regulating trailing growth, pod number per plant, and seed number per plant were assigned. These findings demonstrate the value of the W05 reference genome. The combination of this genomic information and a genetic population that includes wild soybeans will result in the step change required for future soybean breeding.

Several examples demonstrate the unique strength of the W05 reference genome. In partcular, an inversion associated with seed coat color was identified at the *I* locus by comparing W05 with Wm82 and ZH13 genomes. The PCR results and OM data presented here demonstrate that the inversion is the source of PTGS (Figs 2, 3a). Furthermore, major structural changes were identified between the W05 genome and those of two cultivated soybeans. One example is the reciprocal translocation between chromosomes 11 and 13 (Supplementary Figures 3 and 4, Supplementary Table 11). Our findings successfully identify the translocation breakpoints, refining previous results obtained using FISH technology^28^. Together with the high base-to-base fidelity, the W05 genome provides an important tool for future investigations of the variations in genes and alleles in wild soybeans, which may not exist in modern cultivars due to domestication bottlenecks and poorly informed selection criteria^9–13^.

TEs are major drivers of plant evolution^27^. They occupy more than half of the genome sequences of many crop species^27^. Accession-specific TE insertion events can be effectively identified by comparing de novo genome assembles, as repeated regions could cause ambiguities in re-sequencing approaches. Comparisons of the W05 genome with those of two cultivated soybeans revealed that genes with TE insertions that are found in the two cultivated soybean genomes are concentrated in metabolic pathways (Supplementary Data 5). Conversely, similar patterns were not observed in W05-specific TE-affected genes. As metabolic changes lead to variations that are important for soybean production and quality, we may speculate that TE-affected metabolic genes were unintentionally selected during domestication. However, more wild and domesticated germplasm should be tested to further explore this hypothesis. The highlighted importance of variations in metabolic genes during domestication is consistent with our previous observations showing that fixed single-nuclotide polymorphisms (SNPs) are concentrated in metabolic genes in the cultivated soybeans, compared with wild soybeans^31^.

The data presented here also demonstrate the value of the W05 reference genome and the optical contigs of other soybean accessions. Together, these results provide a powerful tool that can be used to uncover large structural variations in soybean germplasm collections (Fig. 3). We present evidence showing that the inversion found in the *I* locus (Fig. 3a) and the translocation between Chromosomes 11 and 13 (Fig. 3b) are events that are found in multiple soybean genomes. Moreover, such comparisons reveal accession-specific genomic regions, e.g., a region on chromosome 15 that was previously reported as a feature of the cultivated soybean^7^ is in fact unique to Wm82 (Fig. 3c).

The strategy of mapping optical contigs to high-quality reference genomes will have a significant impact in soybean breeding programs. For example, new cultivars generated from radiation-driven mutations often contain major structural genomic changes and these structural variations could be effectively identified using this approach. This strategy also provides an elegant means to detect copy number variations, which are the basis of a number of phenotypic traits in humans, animals, and plants^32^. We specifically investigated a region containing multiple trypsin inhibitor genes as an example of how the genome can be interrogated to identify genes that influence seed nutritional quality. Trypsin inhibitors are considered to have evolved as a protective measure against herbivores and bacterial infections. Trypsin inhibitor proteins account for 2–6% total protein of soybean seeds, which is the highest value found in a range of legumes^33^, reducing the nutritional value of the beans as food and/or animal feed. The genomic analysis reported here reveals that a genomic region on chromosome 8, which presumably contains a *KTI* gene cluster, has shrunk in cultivated soybeans from China and other countries compared with most of the wild soybeans from China and Korea (Fig. 3d). This finding suggests that domestication may have involved selection for a reduced copy number of *KTI* genes.

In summary, this study provides information regarding the wild soybean that cannot be easily inferred from the reference genomes of the cultivated soybeans. As wild accessions are important genetic resources for crop improvement, the wild soybean genome reported here will be a valuable, if not indispensable, tool for use in a wide range of applications by legume researchers for comparative genomic and evolutionary studies, and soybean breeders for crop improvement programs.

## Methods

### Sample preparation and sequencing

W05 is a Chinese wild soybean accession originally collected in Henan Province. Union (C08) is a cultivated soybean bred in the United States by crossing Williams and SL12, and then backcrossing five times with Williams. W05 and C08 was used to generate a RI population for the identification of important QTLs and the causal gene of a major QTL for salt tolerance^8,9,14^. Information of other soybean accessions used in this study is presented in Supplementary Data 6.

For Illumina sequencing, seeds of W05 were germinated on 0.8% water agar in sterile magenta box at 28 °C in dark. Hypocotyls and radicals were collected 3–4 days after germination and snap frozen in liquid nitrogen. DNA was extracted from the hypocotyls and radicals of young W05 seedlings using DNeasy Plant Mini Kit (Qiagen, Hilden, Germany, Cat. 69104). The DNA sample was sent to BGI-Shenzhen (Shenzhen, China) for library construction and sequencing on the Illumina HiSeq2000 platform (Illumina, San Diego, CA).

For Pacbio sequencing, W05 was grown in greenhouse on regular soil. Trifoliate leaves were collected and snap frozen in liquid nitrogen. Nuclei were isolated from trifoliate leaves of W05 following a published protocol^34^ with minor modification. Frozen leaves were ground in liquid nitrogen. The powder was suspended in 200 mL nuclei isolation buffer (10 mM Tris HCl pH9.5, 10 mM EDTA, 100 mM KCl, 500 mM sucrose, 4 mM spermidine, 1 mM spermine, and 0.2% (v/v) β-mercaptoethanol) with 0.6% (v/v) Triton X-100 and filtered through 41 nm and 20 nm nylon mesh sequentially. The lysate was centrifuged at 1200 × *g* for 10 min at 4 °C to collect the nuclei. The nuclei was washed twice with nuclei isolation buffer with Triton X-100 and once with nuclei isolation buffer only. Nuclei DNA was extracted using modified cetyltrimethylammonium bromide (CTAB) method^35^. The DNA sample was sent to BGI-Shenzhen and Wuhan Institute of Biotechnology (Wuhan, HuBei, China) for library construction and sequencing. In total, 6 SMRTbell libraries with size selection using BluePippin (Sage Science, Beverly, MA) were constructed and sequenced using 72 SMRT cells with P6-C4 chemistry on PacBio RS II platform (Pacific Biosciences, Menlo Park, CA).

The Dovetail Hi-C library was prepared and sequenced by Dovetail Genomics (Santa Cruz, CA)^36^. Briefly, chromatin in the nucleus of soybean young seedlings was fixed with formaldehyde and extracted. Fixed chromatin was digested with DpnII and sticky ends were filled in with biotinylated nucleotides and ligated. Crosslinks were then reversed and DNA was purified. Purified DNA was treated to remove biotin that was not internal to ligated fragments. The DNA was then sheared to ~350 bp and sequencing libraries were constructed using NEBNext® Ultra™ DNA Library Prep Kit (Illumina, Cat. E7370S). Biotin-containing fragments were enriched through streptavidin bead pulldown before PCR amplification of the library. The library was sequenced on Illumina HiSeq X platform (Illumina).

For Bionano OM, young leaves of all the soybean germplasm used in this study were collected from 7- to 10-day-old seedlings grown in greenhouse and high-molecular weight (HMW) DNA was extracted following the Bionano IrysPrep® High Polysaccharides Plant Tissue DNA Isolation User Guide (Bionano Document Number: 30128). Extracted HMW DNA molecules were fluorescently stained using Nick-Label-Repair-Stain (NLRS) enzymatic reactions following the Bionano Prep™ Labeling - NLRS Protocol (Bionano Document Number: 30024). Briefly, single-strand breaks were introduced to DNA molecules by nicking enzyme Nb.BssSI or Nt.BspQI to generate sequence motif-specific patterns. Nicked sites were labeled with fluorescent nucleotides and repaired. Molecule backbones were also fluorescently stained with YOYO-1 to visualize the full lengths.

NLRS reaction products were then run on Bionano Saphyr (Bionano Genomics, San Diego, CA) for W05 or Irys system (Bionano Genomics) for the rest of the soybean germplasms, where DNA molecules were automatically stretched and imaged within nanochannel arrays. Distances between fluorescently labeled nicking sites form patterns as basis for alignment and assembly by Bionano AutoDetect software (v2.1.4).

Embryos of W05 were collected 24 h after germination in distilled water, in a dark incubator. Cotyledons and hypocotyl were collected 3 days, whereas root, apical buds, and stems were collected 10 days after sowing in vermiculite with 70% water content and growing in a greenhouse. Flower, 7-day pods, 14-day pods, 14-day seeds, 40-day pods, and 40-day seeds were collected at reproductive stages from soybean plant grown in the greenhouse. Total RNA was extracted using RNAiso Plus reagent (TaKaRa, Kyoto, Japan, Cat. 9108). Nodules and remaining roots were collected 28 days post inoculation with *Sinorhizobium fredii* strain CCBAU45436^37^. Total RNA was extracted from nodules and the remaining roots were extracted using TRIzol® reagent (Thermo Fisher Scientific, Waltham, MA, Cat. 15596018).

RNA samples were sent out to BGI-Shenzhen for both RNA sequencing (RNA-seq) and PacBio IsoSeq. For RNA-seq, stranded RNA-seq libraries were constructed for each RNA sample and sequenced on Illumina HiSeq4000 platform (Illumina). For PacBio IsoSeq, equal amount of RNA from different tissues were pooled. Four SMRTbell libraries were constructed with size selection of 1–2 Kb, 2–3 Kb, 3–6 Kb, and 5–10 Kb using the BluePippin (Sage Science), and sequenced with 4, 4, 2, 2 SMRT cells with P6-C4 chemistry on PacBio RS II platform (Pacific Biosciences).

### **De novo** genome assembly

Before de novo assembly, low-quality PacBio subreads with a read length shorter than 500 bp or a quality score lower than 0.8 were filtered out. The remaining clean PacBio subreads were error-corrected and assembled into contigs with MECAT^38^ (version 1.0). The MECAT de novo assembler is composed of three major steps: (i) mecat2pw: all vs. all alignment between PacBio subreads were performed; (ii) mecat2cns: high-quality consensus reads were generated with parameter setting: -a 2000 -c 4 -l 2000; and (iii) mecat2canu: 40 × longest high-quality consensus reads were assembled into primary contigs with parameter setting: genomeSize = 1100000000 ErrorRate = 0.013.

All clean PacBio subreads were mapped to the assembled contigs using pbalign (SMRTLink package release 4.0.0.190159). The primary contigs were polished with mapped PacBio subreads using Quiver implementation in variantCaller (SMRTLink package release 4.0.0.190159).

Illumina short reads with insert size of 250, 500, and 800 bp were used to correct residual errors in the polished contigs. Reads contain adapter sequences or 5% Ns, or with low quality, or derived from PCR artifacts were filtered.

Remaining clean reads were mapped to polished contigs using Burrows-Wheeler Aligner (BWA) mem^39^ (version 0.7.15-r1142) with default parameters. Duplicated reads were tagged using Picard MarkDuplicates implementation (https://broadinstitute.github.io/picard/, version 2.9.0–1-gf5b9f50), with default parameters. Residual errors in the polished contigs were corrected with mapped next generation sequencing (NGS) reads using Pilon^40^ (version 1.22), with the following parameter setting: –diploid–fix snps,indels,local. Polished contigs were used as input for scaffolding analysis.

For scaffolding, HiRise^TM^ pipeline (Dovetail Genomics) was used for scaffolding^41^. Hi-C reads and NGS clean reads with insert size of 250, 500, and 800 bp were mapped to polished contigs using modified Scalable Nucleotide Alignment Program (SNAP) mapper (http://snap.cs.berkeley.edu). Repeats in the contigs were masked, based on NGS read mapping depth. The order and orientation of contigs within Hi-C scaffolds were determined based on contact frequency calculated from mapped Hi-C read pairs.

Low-quality optical molecules with molecule length < 150 Kb or molecule signal intensity > 0.6, or molecule label number < 9 were removed. Optical molecules digested with Nt.BspQI or Nb.BssSI were de novo assembled into optical contigs using Bionano Solve^TM^ (v3.0.1 release v06082017, parameter settings: Iteration:5; minlen:150 kb; minsites:9; initialAssembly: 1.00E − 10; extendRefine: 1.00E − 11; merge: 1.00E − 15). Two-enzyme hybrid scaffolding were performed using Bionano Solve^TM^ (v3.0.1 release v06082017, default parameters) with polished contig sequences and two set of de novo assembled optical contigs as input to generate hybrid scaffolds.

Superscaffolds were generated by merging Hi-C scaffolds and hybrid scaffolds. Linkages between adjacent contigs within superscaffolds were classified into three categories based on supporting evidence: (i) both: linkage supported by both Hi-C scaffolds and hybrid scaffolds; (ii) map: linkage supported only by hybrid scaffolds; and (iii) Hi-C: linkage supported only by Hi-C scaffolds.

To fill and close gaps, PacBio subreads were mapped to superscaffolds using BLASR^42^ (version 1.3.1.142244). Gaps within the superscaffolds were filled with consensus sequences generated from PacBio subreads that span or flank gaps using PBJelly2^43^ (PBSuite_15.8.24). After gap filling, another round of polishing was performed with PacBio subreads and Illumina short reads to eliminate sequence errors in the filled sequences.

Gaps within the superscaffolds were closed by searching for gap-flanking sequences that were identical to each other in a head-to-tail way using BLAST^44^ (version 2.2.31, default parameter). Illumina mate-pair reads with insert size of 2, 6, and 10 Kb, and optical molecules were used to distinguish redundant sequences from true tandem repeated sequences. Illumina mate-pair reads with different insert sizes were mapped to superscaffolds with BWA^45^ aln implementation (version 0.7.15-r1142, parameter settings: -a 50000). Optical molecules were aligned to the in silico digested map of superscaffolds with OMBlast^46^ (version 1.4a) and visualized with OMTools^47^ (version 1.4a). For gaps with identical flanking sequences, insert size for mate-pair reads and signal pattern for optical molecules that span or flank these gaps were manually inspected, to identify redundant sequences that were then trimmed to close the gaps. Another round of sequence polishing was performed, with Illumina short reads to eliminate residual errors.

### Assembly evaluation

The gene completeness of soybean assemblies was evaluated at the contig level using both the PacBio IsoSeq full-length reads and the 1440 conserved BUSCOs. PacBio IsoSeq full-length reads were mapped to genome assemblies using BLAT^48^ (version 35) with default parameters. Only one best hit was retained for each query sequence. The BLAT alignment hits were further filtered based on mapping identity ( ≥95%) and query coverage (≥50% or ≥90%). BUSCO^16^ (version 3.0.2, lineage dataset embryophyta_odb9) was used to identify conserved BUSCO genes in the genome assemblies. Telomeric repeats were identified by tandem repeat finder (TRF)^49^ (version 4.0.4). Centromeric repeats were identified by BLAST^44^ (version 2.2.31, *E*-value < 1e − 5) search Cent91/92 sequences against the genome sequence^17^. Primer sequences of soybean genetic map were downloaded from SoyBase (https://www.soybase.org/dlpages/#geneticmap). In silico PCR was performed using isPCR (https://github.com/bowhan/kent/tree/master/src/isPcr/isPcr), e-PCR^50^ (version 2.3.9, parameters: D = 30–1000 N = 2 G = 2 T = 4), and BLAST^44^ (version 2.2.31, *E*-value < 1e − 5). Only primer pairs that mapped to the genome with proper orientation (forward-reverse or reverse-forward) and proper insert size (< 1000 bp) were retained. Primer pairs that have multiple best hits in the genome were filtered.

### Repeat annotation

Tandem repeats in the genome assembly was identified using TRF^49^ (version 4.0.4). Well-characterized TEs were identified by searching against W05 genome assembly at DNA level and protein level using RepeatMasker (version 4.0.7) (http://www.repeatmasker.org/) and ProteinRepeatMask (version 4.0.7) (http://www.repeatmasker.org/) with RepBase^51^ (release 20.04) as the query library. To identify TEs that were absent in the RepBase library, a de novo repeat library was constructed using Repeatmodeler (version 1.0.10) (http://www.repeatmasker.org/). RepeatMasker was run against the genome assembly again, with de novo repeat library as the query library.

### Gene annotation

For homology-based evidence generation, the sequences of proteins from *Arabidopsis thaliana* (araport11), *Lotus japonicas* (3.0, ftp://ftp.kazusa.or.jp/pub/lotus/lotus_r3.0/), *G. max* (Phytozome release 12), *Vitis vinifera* (Phytozome release 12), *Medicago truncatula* (Phytozome release 12), *Prunus persica* (Phytozome release 12), and *Populus trichocarpa* (Phytozome release 12) were downloaded. For each species, only the longest protein sequences were retained as the representative for each gene locus. The representative protein sequences were mapped to *G. soja* W05 genome using splice-site-aware aligner Exonerate^52^ (version 2.4.0) with the following parameters: –model protein2genome–showalignment–showtargetgff–refine region. Only the best alignment with the highest score was retained for each mapped gene locus for each species.

For expression-based evidence generation, low-quality RNA-seq reads were filtered and trimmed using trim_galore (version 0.4.1) (http://www.bioinformatics.babraham.ac.uk/projects/trim_galore/). Trimmed reads with read length shorter than 80 bp were discarded. Stranded and non-stranded RNA-seq reads were assembled into stranded and non-stranded unigenes using Trinity^53^ (version 2.4.0) with default parameters, respectively. The assembled stranded and non-stranded unigenes were mapped to genome sequences and assembled into non-redundant transcripts using PASApipeline^54^ (version 2.2.0) with the following parameter setting: –MAX_INTRON_LENGTH 20000. Furthermore, consensus PacBio IsoSeq subreads were extracted, classified, and clustered to generate corrected consensus reads using SMRTanalysis package (smrtanalysis_2.3.0.140936). Corrected consensus sequences with low-quality score were further corrected with stranded RNA-seq reads using proovread^55^ (version 2.13.12, parameter setting: –mode sr). After correction, the consensus sequences were mapped to the genome assembly and assembled into non-redundant transcripts using PASApipeline^54^ (version 2.2.0) with parameters: –MAX_INTRON_LENGTH 20000.

For evidence synthesis and alternative spliced isoform annotation, transcripts that were well supported by both homology-based evidence and expression-based evidence were selected for parameter training for ab initio prediction software Augustus^56^ (version 3.2.3). All the generated transcript evidences mentioned above and trained Augustus parameters were fed into Maker^57^ (version 2.31.9) for evidence synthesis to generate primary protein-coding gene set. The primary gene set and expression-data-based evidence were fed into PASApipeline^54^ (version 2.2.0) for gene structure refinement and alternative spliced isoform annotation.

Potential function was assigned to each annotated protein sequence using InterProScan^58^ (version 5.29–68.0) by searching all available databases with corresponding utilities. Completeness of the annotated gene set was evaluated by BUSCO^16^ (version 3.0.2, lineage dataset embryophyta_odb9).

### ncRNA annotation

rRNA sequences of *G. max* were downloaded from NCBI GenBank database (GenBank ID: X15199.1, AJ009787.1, X02623.1, and AH001766.2). These rRNA sequences were mapped to *G. soja* W05 genome assembly using BLAST^44^ (version 2.2.31) with default parameters.

tRNAScan-SE^59^ (version 1.3.1) was used to search the *G. soja* W05 genome assembly for tRNAs genes with default parameters. Annotated tRNA genes that were classified as Pseudo were filtered.

To identify miRNA and snRNA genes, infernal^60^ (version 1.1.2, parameters: –cut_ga–rfam–nohmmonly) was used to search the *G. soja* W05 genome assembly based on covariance models deposited in Rfam^61^ database (release 13.0).

### Binmap construction and QTL identification

Binmap construction and QTL identification was performed with population sequencing and phenotype datasets generated in our previous studies^8^ using a slightly modified analysis pipeline. Briefly, short reads from the 96 RI lines were mapped to the genome sequence using BWA^45^ (version 0.7.15-r1140) with default parameters. Properly and uniquely mapped reads were extracted to identify SNPs using Samtools^62^ (version 1.2) with parameter setting: SNP quality value > 30 and < 3 SNPs were allowed in any 10 bp window. Heterozygous or non- parental line-derived SNPs were filtered. At least 20 RI lines were required to have SNPs at each SNP locus^8^. Recombination breakpoints were identified using a modified sliding window approach (window size: 15 SNPs, step size: 1 SNP)^63^. Adjacent 50 Kb intervals were merged into bins if no recombination events were identified from sequenced RI lines. Genetic distance among bins was calculated using R/qtl package^64^ (version 1.41.6, default parameters). QTLs for each agronomical trait were identified using QTL Cartographer (http://statgen.ncsu.edu/qtlcart/) (version 1.17j) with a 10 cM scanning window and a 0.5 cM step size. For each agronomical trait, logarithm of the odds (LOD) cutoff was determined by 1000 permutation of the phenotypes and genotypes with significance level of *α* < 0.05 using QTL Cartographer. Peaks having LOD value higher than the LOD cutoff were considered as significant. For green seed phenotype dataset, only RI lines of the green and yellow seeds were used. For seed size phenotype dataset, about 35 seeds from each RI line were measured using SmartGrain^65^ (version 1.1).

For the same QTL for each agronomical trait, sequence alignment was performed between two QTL regions (with referenced to different genomes) using the nucmer program from MUMmer package^66^ (version 4.0), with default parameter. Aligned block with length smaller than 1 Kb were filtered. The overall aligned region was defined as overlapped region.

### Genome-wide TE insertion identification

Repeat elements in cultivated soybean genomes were annotated using the same pipeline as used for W05 genome repeat annotation. To identify TE insertions in Wm82_v2 and ZH13 genomes, the two genomes were aligned to W05. In reverse, the W05 genome was compared with those of Wm82 and ZH13 for W05-specific insertions. Genome comparisons were performed using nucmer from MUMmer package^66^ (version 4.0) with parameters: –mum–noextend. Adjacent alignment blocks with a gap length > 1000 bp in query genome and <100 bp in reference genome were identified as insertions in the query genome. If >80% of the inserted regions in the query genome were annotated as TE elements, the insertion was defined as a TE insertion and the corresponding alignment gap in the reference genome was defined as a TE insertion site. If a TE insertion site in the reference genome was located within a genic region or a 500 bp flanking genic region, then genes were defined as TE-affected genes in the query genome. GO enrichment analysis was performed for TE-affected genes using BINGO (version 3.0.3)^67^.

### Whole-genome sequence comparison

Whole-genome comparison between wild soybean W05 and two cultivated soybean genomes were performed using nucmer from MUMmer package^66^ (version 4.0) and visualized with mummerplot from MUMmer package (version 4.0). Large structural variations (>100 Kb) were identified based on synteny alignment blocks, with variation boundaries manually checked.

### Optical map analysis of multiple soybean accessions

To enhance result quality, quality assessment using DataQualityCheck module^47^ was performed to exclude optical molecules from scans with possible anomalies. Throughput, signal-to-noise ratios and alignment rates in datasets were indentified using a reference in silico map. They were checked for deviations from the norm, which may indicate clogging of the nanochannel arrays.

OM de novo assembly was performed as follows. Filtered optical molecules were assembled into optical contigs using Bionano Solve (version 3.1) assembly pipeline (pipelineCL.py), with parameters optimized by Bionano (optArguments_nonhaplotype_irys.xml; -minlen 150, -minsites 9; merge: 1.00E − 15), with *p*-value cutoff thresholds for initialAssembly and extendRefine modified according to genome size to 1E − 8 and 1E − 9, respectively.

For better accuracy in downstream analysis, assembled optical contigs were preprocessed with the OMTools DataTools module before alignment. In consideration of the resolution of signal detection, signals within 1 Kb apart were merged for both optical molecules and optical contigs to improve alignment accuracy at regions of dense signals. Optical contigs containing only repetitive segments were removed by OMTools (version 1.4a) DataTools module with the lowden parameter. Processed optical molecules were aligned to retained optical contigs with OMblast (version 1.4a)^46^. Optical contigs with optical molecule coverage lower than 30 at the target region were excluded from subsequent comparative analysis.

To locate the coordinates of the optical contigs, sequence assemblies of W05, Wm82, and ZH13 were in silico digested for alignment. Signals on the in silico maps within 1 Kb were merged. For each region of interest, optical contigs or in silico maps were aligned to the W05 reference in silico map with OMBlast^46^. Pairwise alignment was performed between optical contigs that aligned to the target region. Multiple alignment of the optical contigs was then performed to obtain linkage information of genome segments using Optical Map Multiple Alignment package (https://github.com/TF-Chan-Lab/OMTools)^47^ (version 1.4a). Signal patterns and linkage information were compared, to characterize genomic structural variations among germplasms.

### PCR verification of the inversion junction at the *I* locus

To verify the inversion in the *I* locus of soybeans with colorless seed coat, junction of the inversion that created the subtilisin-anti-*CHS1* chimeric transcript were amplified from genomic DNA of selected soybean accessions. For the detection of subtilisin-anti-*CHS1* chimeric transcript, RNA was extracted from the seed coat of developing seeds of Wm82 and W05 using Fruit-mate^TM^ for RNA purification (Takara, Cat. 9192) and RNAiso Plus reagent (Takara, Cat. 9108). First-strand cDNAs and subsequent PCR were done using One-Step TB Green™ PrimeScript™ RT-PCR Kit II (Takara, Cat. RR086). In the reverse-transcription step, only the reverse primer specific for either the subtilisin-anti-*CHS1* chimeric transcript or the *GmACT11* gene^68^ (housekeeping gene) was added. Forward primers were then added for the subsequent PCR amplification. Primer information can be found in Supplementary Table 12.

### Hi-C contact frequency calculation

Hi-C raw reads were mapped to W05 genome and Hi-C contact frequency between genomic loci was computed using Juicer^69^ (version 1.5) with window size of 100 Kb. The Hi-C reads contact frequency matrix was visualized using Juicebox^70^ (version 1.5.2).

### Reporting summary

Further information on experimental design is available in the Nature Research Reporting Summary linked to this article.

## Supplementary information

Supplementary Information

Peer Review

Reporting Summary

Description of Additional Supplementary Data

Supplementary Data 1

Supplementary Data 2

Supplementary Data 3

Supplementary Data 4

Supplementary Data 5

Supplementary Data 6

Source Data

## Data Availability

Genome assembly and annotations data of *G. soja* W05 were deposited in the DDBJ/ENA/GenBank under accession QZWG00000000 [https://www.ncbi.nlm.nih.gov/assembly/GCA_004193775.1/]. The version described here is version QZWG01000000. All the raw sequencing reads were deposited in the NCBI Sequence Read Archive database under the accession SRP158454. The optical molecules and optical contigs of W05 were deposited as NCBI Supplementary Files under accession SUPPF_0000002760 [ftp://ftp.ncbi.nlm.nih.gov/pub/supplementary_data/bionanomaps.csv] and SUPPF_0000002761 [ftp://ftp.ncbi.nlm.nih.gov/pub/supplementary_data/bionanomaps.csv]. The optical contigs of other soybean accessions were deposited as NCBI Supplementary Files under accessions SUPPF_0000002797-SUPPF_0000002807 [ftp://ftp.ncbi.nlm.nih.gov/pub/supplementary_data/bionanomaps.csv]. W05 genome assembly and annotation are also available at wildsoydb database (www.wildsoydb.org/Gsoja_W05). Seeds of the sequenced wild soybean accession W05 cannot be freely distributed to scientists outside of China due to legal restrictions on the exchange of wild plant germplasms. However, seeds of the other parental line C08 and the derived recombinant inbreeding lines are available from Hon-Ming Lam (honming@cuhk.edu.hk) upon request. Data supporting the findings of this work are available within the paper and its Supplementary Information files. A reporting summary for this article is available as a Supplementary Information file. The datasets generated and analyzed during the current study are available from the corresponding author on reasonable request. The source data for Fig. 2b and Supplementary Figure 6 are provided as a Source Data file.
