# Supplementary material for: A reference-grade wild soybean genome
Source: Nat Commun. 2019 Mar 14;10:1216. doi: 10.1038/s41467-019-09142-9 (PMC6418295; doi:10.1038/s41467-019-09142-9)
Supplement: Supplementary file 1 — Supplementary Information [file 41467_2019_9142_MOESM1_ESM.pdf]

# Supplementary Information for

# A reference-grade wild soybean genome

Xie *et al*.

## Table of Contents

Supplementaty Figures ... 3

Supplementary Figure 1 ... 3

Supplementary Figure 2 ... 5

Supplementary Figure 3 ... 6

Supplementary Figure 4 ... 8

Supplementary Figure 5 ... 10

Supplementary Figure 6 ... 11

Supplementary Figure 7 ... 12

Supplementary Tables ... 13

Supplementary Table 1 ... 13

Supplementary Table 2 ... 14

Supplementary Table 3 ... 15

Supplementary Table 4 ... 16

Supplementary Table 5 ... 17

Supplementary Table 6 ... 18

Supplementary Table 7 ... 19

Supplementary Table 8 ... 20

Supplementary Table 9 ... 21

Supplementary Table 10 ... 22

Supplementary Table 11 ... 23

Supplementary Table 12 ... 24

References ... 25

## Supplementaty Figures

## Supplementary Figure 1

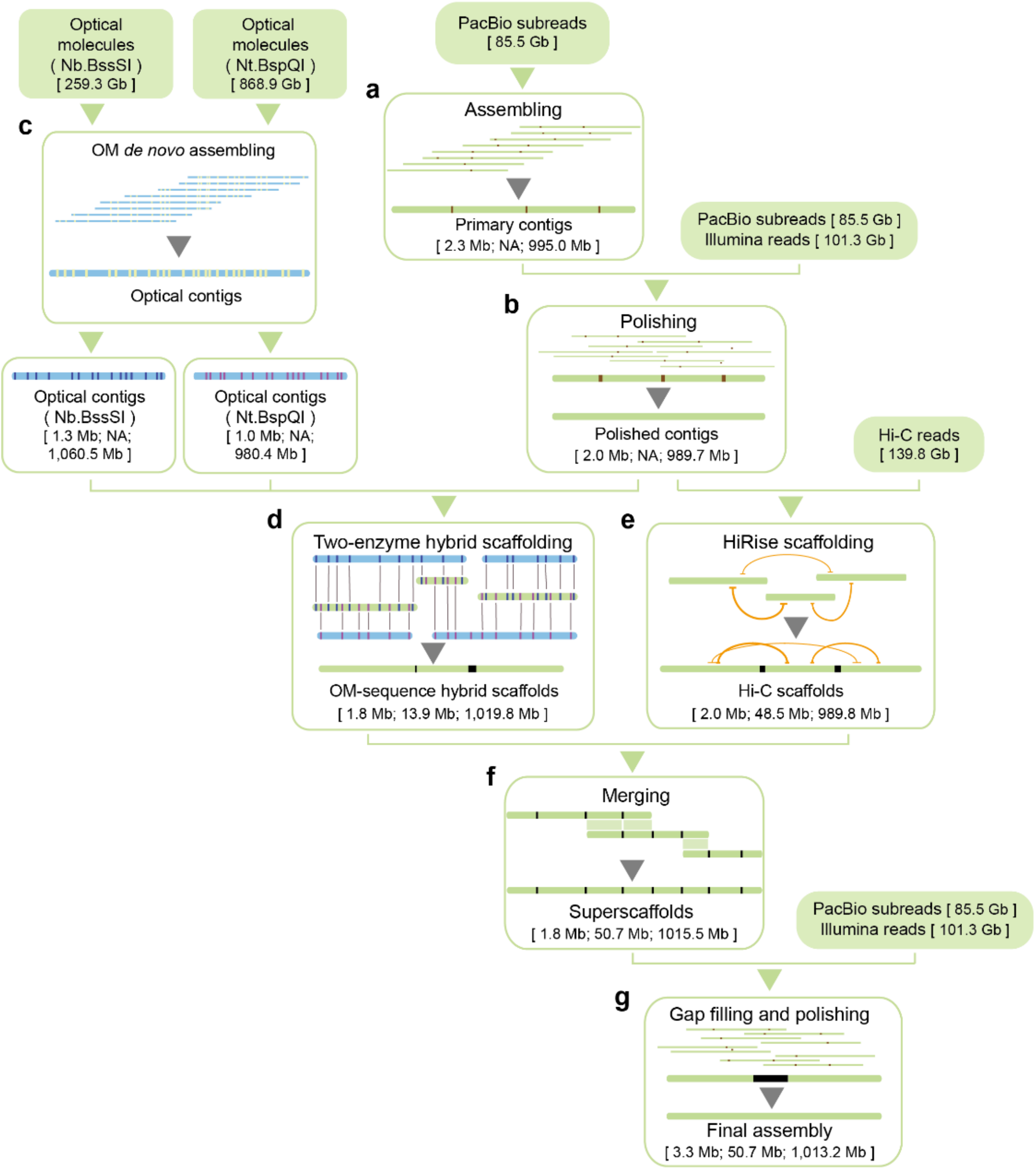

## Supplementary Figure 1

**Supplementary Figure 1. Work flow and summary of the *de novo* assembly process. (a)** PacBio subreads were assembled into primary contigs; and **(b)** polished with PacBio subreads and Illumina reads sequentially. **(c)** Two sets of optical molecules were generated using different enzymes, and then assembled into optical contigs, respectively. **(d)** Polished contigs were ordered and oriented into scaffolds based on *de novo* assembled optical contigs; and **(e)** Hi-C contact frequency independently. **(f)** The two scaffold sets were merged to generate superscaffolds. **(g)** Gaps within superscaffolds were filled with PacBio subreads and then polished with PacBio subreads and Illumina reads again to get the final assembly. Errors and gaps in sequences are illustrated as brown and black blocks, respectively. Input data are shown in rounded rectangle with green background color, while analysis procedures are shown in rounded rectangle with white background. Input data amount and assembly statistics, in the format of: contig N50; scaffold N50; total assembly size, at different stages are shown in square brackets. NA: not applicable. Thickness of the orange lines in HiRise scaffolding step is correlated with the magnitude of Hi-C contact frequency.

## Supplementary Figure 2

**a**

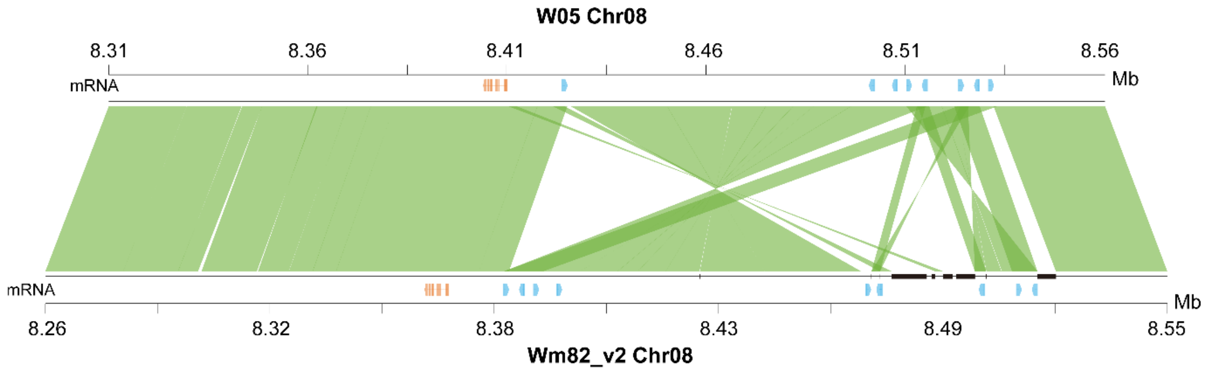


**b**

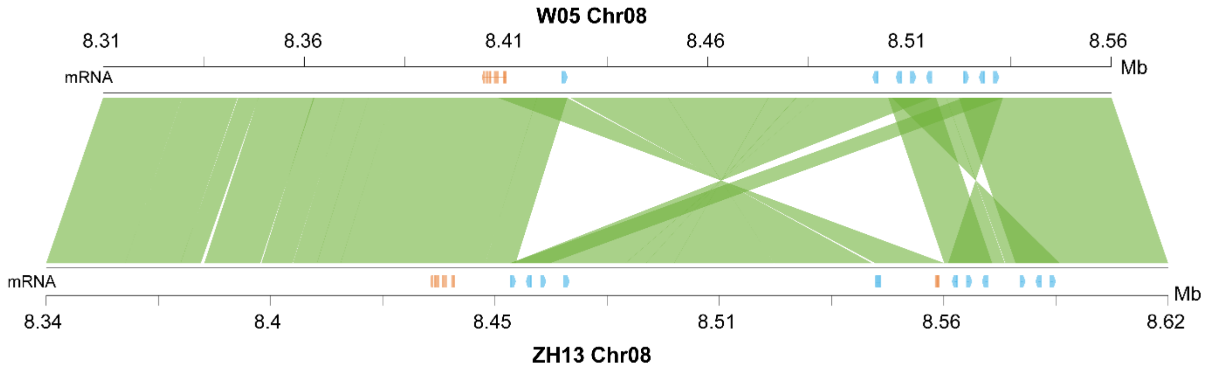


**Supplementary Figure 2. Sequence comparison of *I* locus between different reference genomes. (a)** Sequence comparison between W05 and Wm82_v2. **(b)** Sequence comparison between W05 and ZH13. Blue arrowheads indicate the positions of *CHS* genes. Positions of the original intact and duplicated fragments of the subtilisin gene were indicated in orange. Aligned region is shaded green. Bolded black lines indicate gaps in the genome assemblies.

## Supplementary Figure 3

**a**

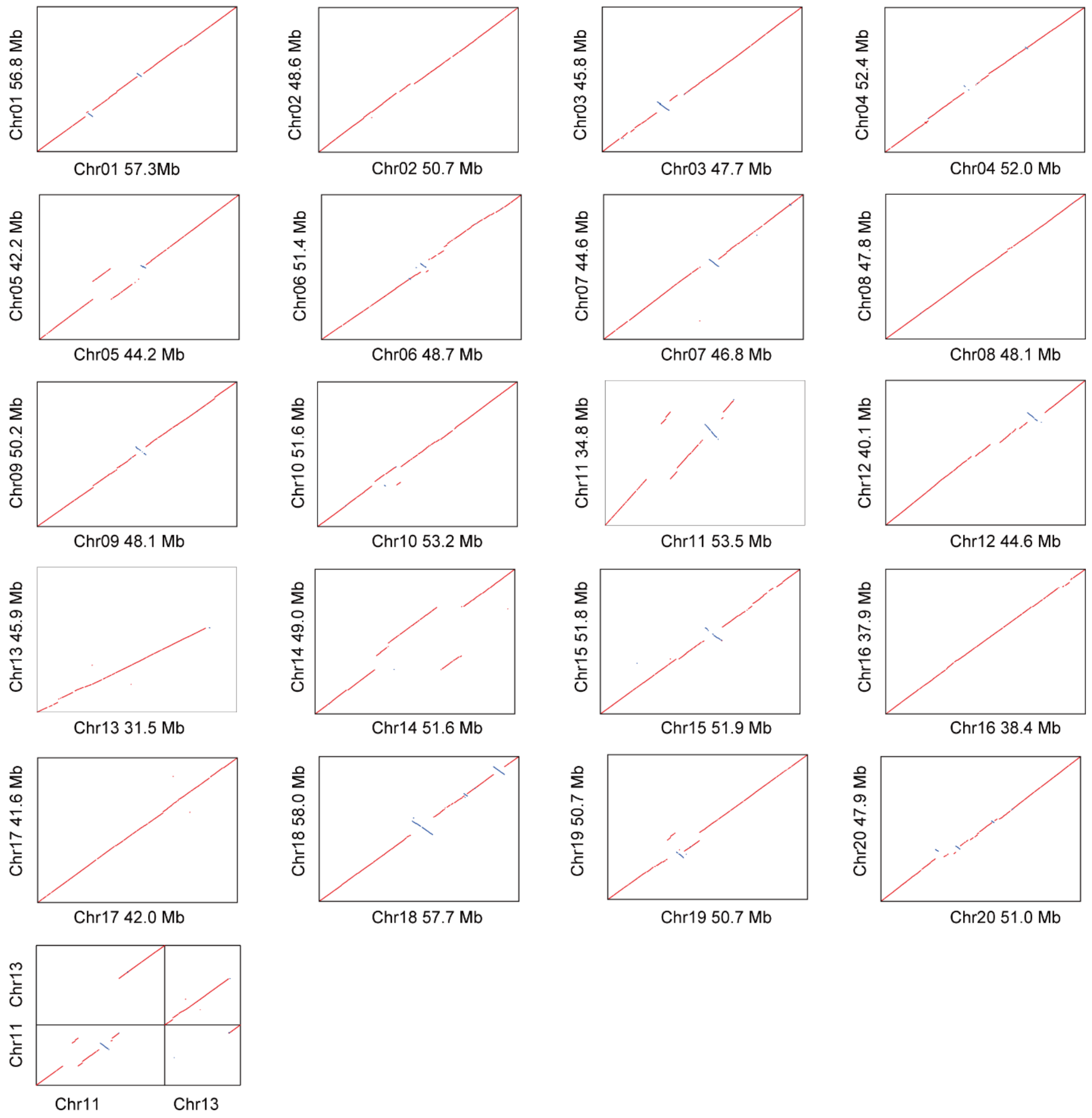

## Supplementary Figure 3 (continued)

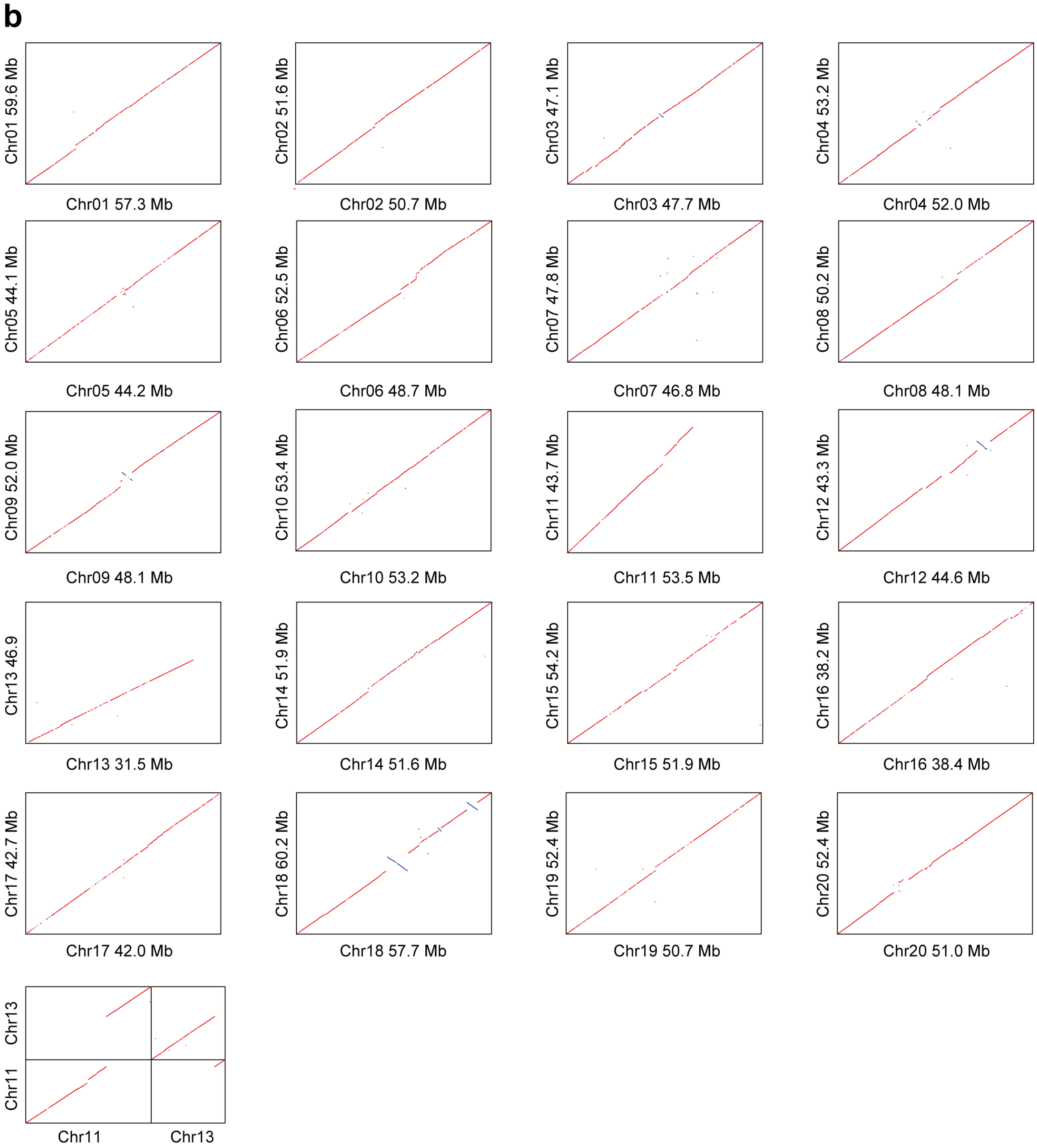


**Supplementary Figure 3. Collinearity plot between genomes.** Collinearity between **(a)** W05 and Wm82_v2, and **(b)** W05 and ZH13 across 20 chromosomes. Red lines show collinear regions with the same orientation while blue lines show collinear region with inversion. X-axis: W05 chromosomes. Y-axis: **(a)** Wm82_v2 or **(b)** ZH13 chromosomes. Bottom left panel shows the collinearity of chromosomes 11 and 13 which indicates inter-chromosomal translocation.

## Supplementary Figure 4

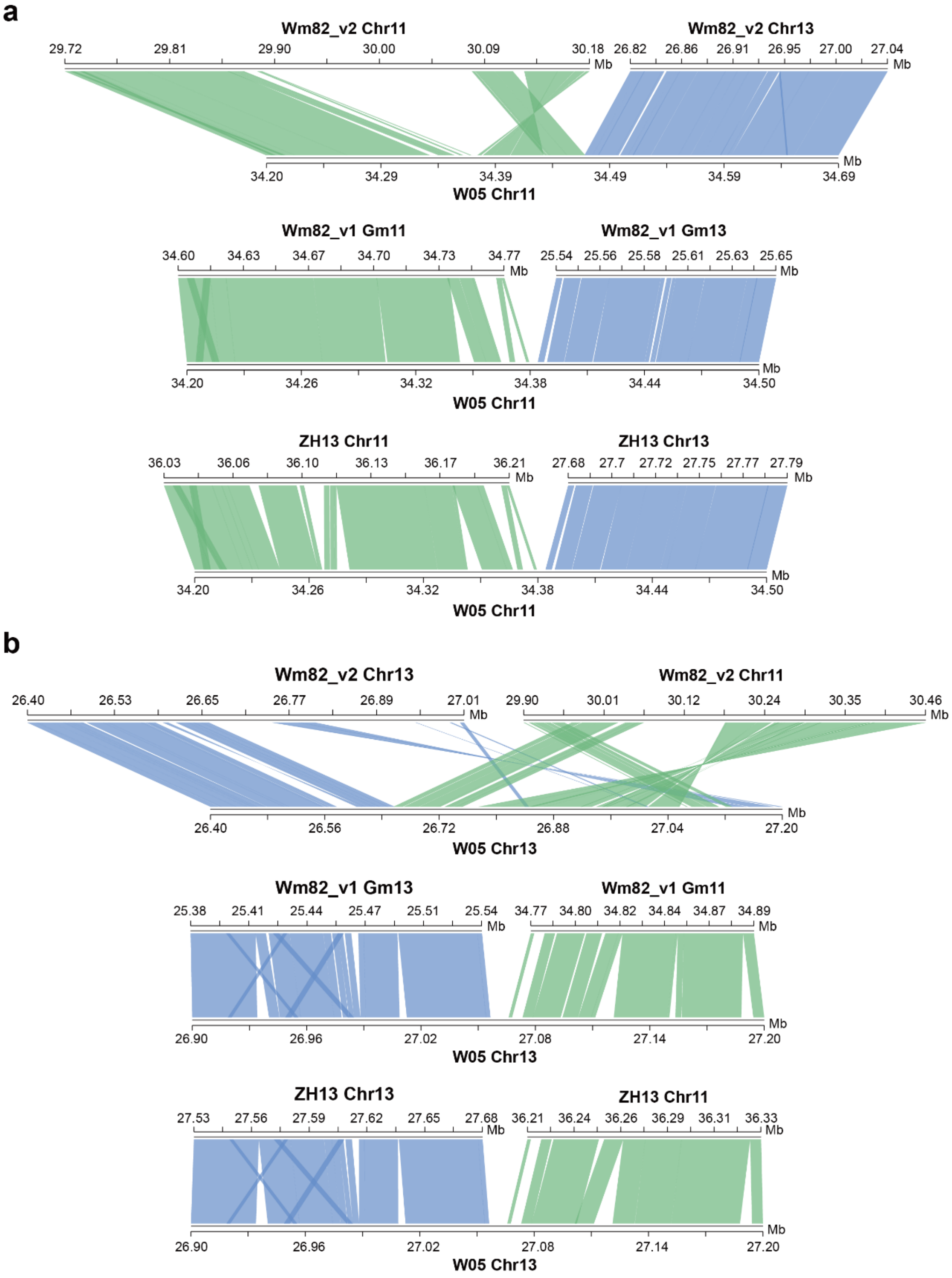

## Supplementary Figure 4

**Supplementary Figure 4. Sequence comparison of the reciprocal inter-chromosomal translocation junctions between chromosomes 11 and 13. (a)** Comparison with reference to W05 Chr11, and **(b)** comparison with reference to W05 Chr13. Green shaded: regions aligned to Chr11 of Wm82 and ZH13; Blue shaded: regions aligned to Chr13 of Wm82 and ZH13.

# Supplementary Figure 5

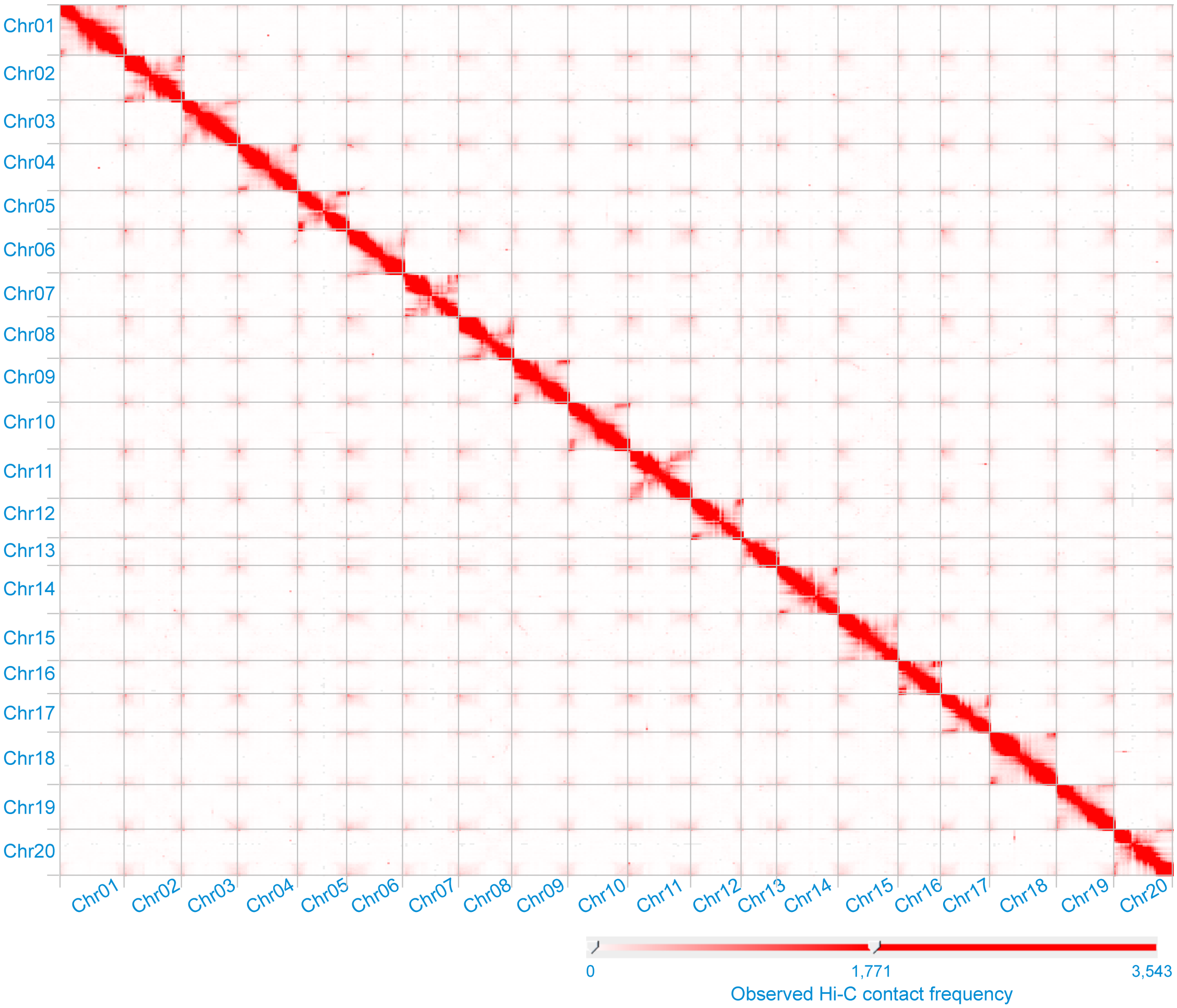


**Supplementary Figure 5. Hi-C reads contact frequency along W05 chromosomes.** Hi-C reads were mapped to W05 genome and Hi-C contact frequency between genomic loci was calculated with a window size of 100 Kb.

## Supplementary Figure 6

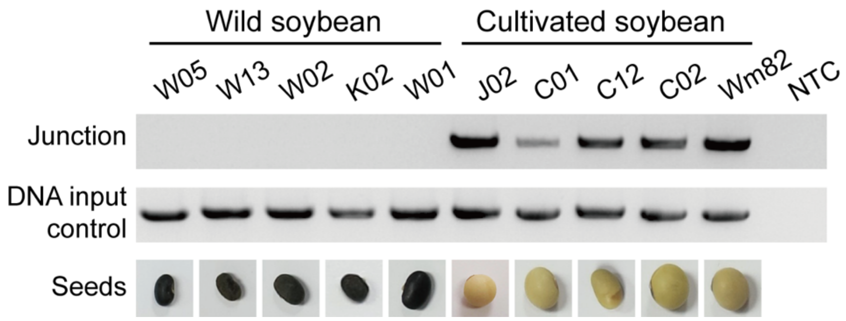


**Supplementary Figure 6. PCR amplification of the junctions of the inversion at *I* locus.** Junction: inversion junction that creates the subtilisin-anti-*CHS1* chimeric transcript; DNA input control: genomic region at the *I* locus that are shared between accessions with pigmented seed coat and colorless seed coat. Photo of the seed of each accession is shown at the bottom to indicate the seed coat color. NTC: no template control. The experiment has been repeated twice with the same results. Unprocessed gel images are provided in Source Data file.

## Supplementary Figure 7

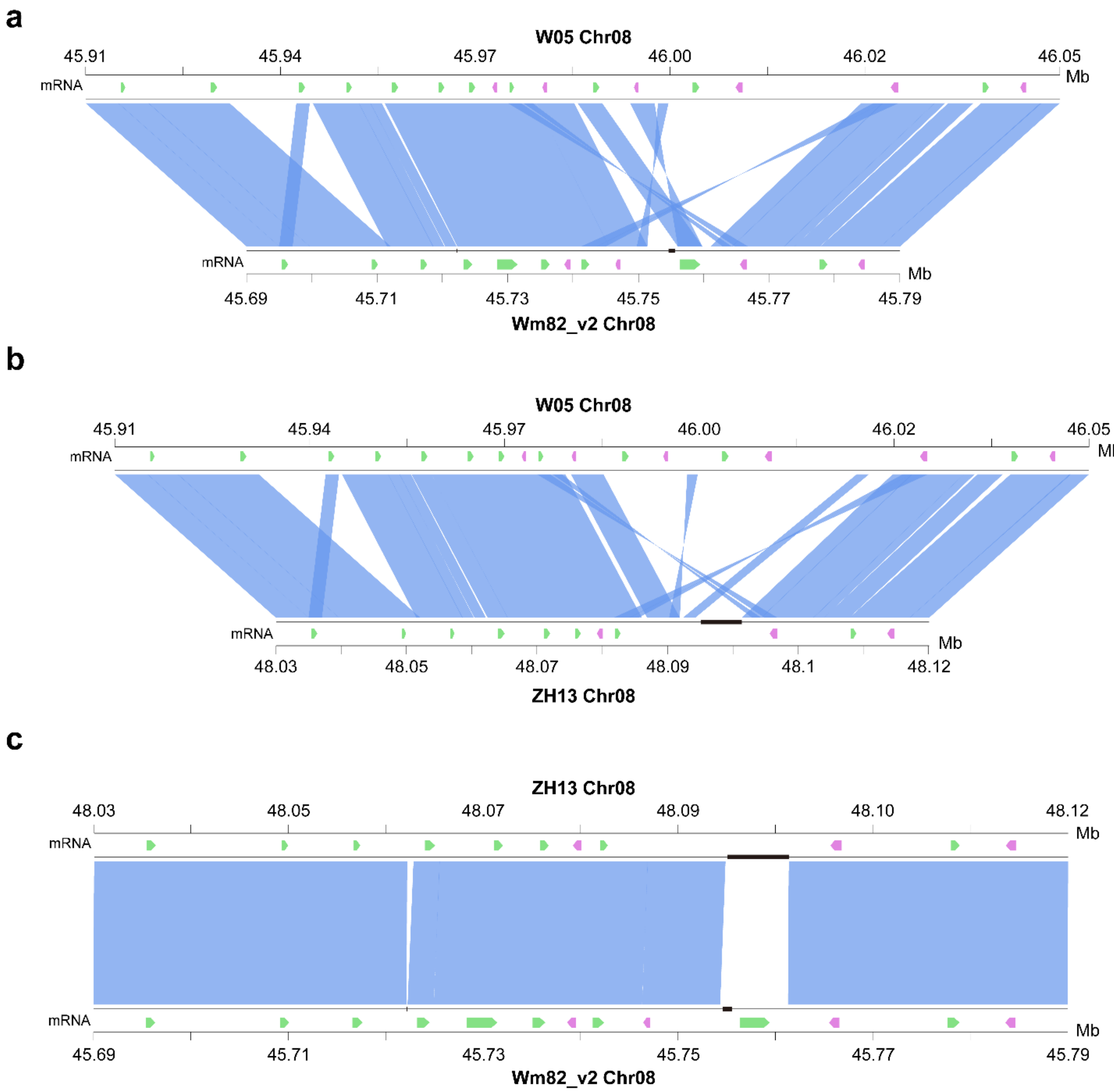


**Supplementary Figure 7. Sequence comparison of the *KTI* gene cluster on chromosome 8.** (**a**) Comparison between W05 and Wm82_v2. (**b**) Comparison between W05 and ZH13. (**c**) Comparison between ZH13 and Wm82_v2. Aligned regions are shaded blue. Each arrowhead indicates one annotated *KTI* gene. Bolded black lines indicate gaps in the genome assemblies.

# Supplementary Tables

## Supplementary Table 1

**Supplementary Table 1**. Summary of sequencing data used for *de novo* assembly.

| Library type | Insert size (bp) | Read length (bp) | Total length (Gb) | Depth* (X) | Purpose | Note# |
|---|---|---|---|---|---|---|
| NGS paired-end | 250 | 150 | 39.5 | 36 | Assembly polishing | |
| | 500 | 100 | 37.3 | 33.9 | | |
| | 800 | 100 | 24.5 | 22.3 | | |
| NGS mate-pair | 2000 | 44 | 4.5 | 4.1 | Gap closing | SRR1185933 |
| | 6000 | 44 | 2.5 | 2.3 | | SRR1185935 |
| | 10000 | 44 | 7.1 | 6.5 | | SRR1185936 |
| Dovetail Hi-C | NA | 151 | 139.8 | 127.1 | Scaffolding | |
| PacBio CLR | NA | [500-68,596] | 85.5 | 77.7 | *De novo* assembling | |

*Assuming that the genome size of *Glycine soja* W05 is 1.1 Gb.

# Accession number for sequencing data that were downloaded from NCBI SRA database.

## Supplementary Table 2

**Supplementary Table 2**. Statistics of optical molecules.

| | Raw data | | Clean data | |
|---|---|---|---|---|
| **Nicking endonuclease** | **Nt.BspQI** | **Nb.BssSI** | **Nt.BspQI** | **Nb.BssSI** |
| Number of molecules | 3,354,788 | 10,222,219 | 375,250 | 1,625,936 |
| Total molecule length (Mb) | 259,266.1 | 868,934.7 | 91,144.1 | 429,127.5 |
| Average molecule length (Kb) | 77.3 | 85.0 | 242.9 | 263.9 |
| Molecule length N50 (Kb) | 125.0 | 157.6 | 240.1 | 271.6 |
| Label number per 100 Kb | 9.3 | 15.8 | 9.0 | 13.3 |

# Supplementary Table 3

**Supplementary Table 3.** Summary of soybean genome assemblies

| Accession | Origin | Assembled genome size (Mb) | Contig N50 (Kb) [a] | Scaffold N50 (Kb) | Wild/ Cultivated | Released year | Reference |
|---|---|---|---|---|---|---|---|
| *G. soja* IT182932 | Yong-In City, South Korea | 915 | NA | NA | Wild | 2010 | [1] |
| *G.Soja* A | Zhejiang, China | 813 | 9.0 | 18.3 | Wild | 2014 | [2] |
| *G.Soja* B | Ibaraki, Japan | 895 | 22.2 | 57.2 | Wild | 2014 | [2] |
| *G.Soja* C | Chungchong Puk,Korea | 841 | 8.0 | 17.0 | Wild | 2014 | [2] |
| *G.Soja* D | Shandong, China | 985 | 11.0 | 48.7 | Wild | 2014 | [2] |
| *G.Soja* E | Shanxi, China | 920 | 27.0 | 65.1 | Wild | 2014 | [2] |
| *G.Soja* F | Heilongjiang, China | 886 | 24.3 | 52.4 | Wild | 2014 | [2] |
| *G.Soja* G | Khabarovsk, Russia | 878 | 19.2 | 44.9 | Wild | 2014 | [2] |
| *G. soja* Lanxi 1 | Lower Yangtze region, China | 930 | 21.7 | 51.0 | Wild | 2014 | [3] |
| *G. max* Enrei 2.0 | Japan | 928 | NA | NA | Cultivated | 2015 | [4] |
| *G. max* Wm82 [b] | USA | 978 | 182.8 | 48,577.5 | Cultivated | 2015 | [5] |
| *G. max* ZH13 | China | 1,025 | 3,250.5 [c] | 51,865.5 | Cultivated | 2018 | [6] |
| *G. max* Lee | USA | 991 | 37.7 | 50,397.3 | Cultivated | 2018 | GenBank: GCA_002905335.1 |
| *G. soja* PI483463 | Shanxi, China | 985 | 24.1 | 48,820.3 | Wild | 2018 | GenBank: GCA_002907465.1 |
| *G. soja* W05 | Henan, China | 1,013 | 3,331.2 | 50,696.8 | Wild | This study | This study |

[a] For reference-based genome assemblies, contig N50 and scaffold N50 were not calculated.

[b] Wm82_v2 (*Glycine max* Wm82.a2.v1) is used.

[c] To be comparable with other genome assemblies, contig N50 is calculated based on total contig length instead of total assembly size used in the original paper.

## Supplementary Table 4

**Supplementary Table 4**. Genome assembly completeness evaluation with 1,440 BUSCO groups.

| Genome assembly | Complete (%) | Fragmented (%) | Missing (%) |
|---|---|---|---|
| W05 | 93.1 | 1.3 | 5.6 |
| Wm82_v2 | 92.7 | 1.5 | 5.8 |
| ZH13 | 93 | 1.2 | 5.8 |

## Supplementary Table 5

**Supplementary Table 5**. Mapping statistics of PacBio IsoSeq high quality consensus sequences.

| Assembly | Identity >=0.95 and coverage >=0.50 | | Identity >=0.95 and coverage >=0.90 | |
|---|---|---|---|---|
| | Number | Percent (%) | Number | Percent (%) |
| W05 | 85,361 | 98.4 | 84,794 | 97.7 |
| Wm82_v2 | 84,242 | 97.1 | 81,738 | 94.2 |
| ZH13 | 85,768 | 98.9 | 85,183 | 98.2 |

## Supplementary Table 6

**Supplementary Table 6**. Detection of telomeric repeats in W05, Wm82_v2 and ZH13 genomes.

| Chromosome | W05 | | Wm82_v2 | | ZH13 | |
|---|---|---|---|---|---|---|
| | Chr start repeat# | Chr end repeat# | Chr start repeat# | Chr end repeat# | Chr start repeat# | Chr end repeat# |
| Chr01 | √ | √ | √ | × | √ | √ |
| Chr02 | √ | √ | √ | √ | × | × |
| Chr03 | √ | √ | √ | √ | √ | √ |
| Chr04 | √ | √ | √ | √ | √ | × |
| Chr05 | √ | √ | √ | × | × | × |
| Chr06 | √ | × | √ | × | × | × |
| Chr07 | √ | √ | √ | √ | × | √ |
| Chr08 | √ | × | √ | × | × | × |
| Chr09 | × | √ | √ | √ | √ | √ |
| Chr10 | √ | √ | × | √ | × | √ |
| Chr11 | √ | √ | √ | × | × | × |
| Chr12 | √ | √ | √ | √ | √ | √ |
| Chr13 | × | √ | × | √ | × | × |
| Chr14 | √ | × | √ | × | √ | √ |
| Chr15 | √ | √ | √ | × | × | √ |
| Chr16 | √ | √ | √ | √ | × | √ |
| Chr17 | × | √ | √ | × | × | × |
| Chr18 | √ | √ | √ | √ | √ | × |
| Chr19 | √ | √ | × | × | √ | √ |
| Chr20 | √ | × | √ | √ | √ | × |

# √ Telomeric tandem repeats TTTAGGG/CCCTAAA were detected at the distal end of assembled chromosomes.

× Telomeric tandem repeats were not detected.

## Supplementary Table 7

**Supplementary Table 7**. Statistics of PacBio IsoSeq consensus reads.

| Size selection | No. of read of insert (ROI) | No. of full length non-chimeric ROI |
|---|---|---|
| 1-2 Kb | 370,342 | 205,951 |
| 2-3 Kb | 273,439 | 121,273 |
| 3-6 Kb | 127,226 | 65,868 |
| 5-10 Kb | 56,553 | 21,658 |
| Total | 827,560 | 414,750 |

## Supplementary Table 8

**Supplementary Table 8**. Statistics of genes in wild soybean W05 and cultivated soybeans Wm82_v2 and ZH13.

| Gene set | W05 | Wm82_v2 | ZH13 |
|---|---|---|---|
| Gene loci | 55,539 | 56,044 | 52,051 |
| Transcripts | 89,477 | 88,647 | 58,017 |
| Alternative spliced transcripts | 33,938 | 32,603 | 5,966 |
| Average transcript length (bp) | 1,955 | 1,838 | 1,647 |
| Average exon number | 6.46 | 6.46 | 5.95 |
| Average exon length (bp) | 303 | 285 | 277 |
| Average length of coding sequences (bp) | 1,342 | 1,275 | 1,342 |
| Average CDS number per transcript | 5.97 | 5.93 | 5.75 |
| Average CDS length (bp) | 225 | 215 | 233 |
| No. of transcripts with 5’-UTR | 69,455 | 73,366 | 26,846 |
| No. of transcripts with 3’-UTR | 71,271 | 74,625 | 29,627 |
| Single exon transcripts | 9,325 | 9,717 | 7,178 |

## Supplementary Table 9

**Supplementary Table 9.** Gene set completeness evaluation with 1,440 BUSCO groups.

| Gene set | Complete (%) | Fragmented (%) | Missing (%) |
|---|---|---|---|
| W05 | 97.5 | 0.5 | 2 |
| Wm82_v2 | 97.3 | 0.6 | 2.1 |
| ZH13 | 97 | 0.8 | 2.2 |

## Supplementary Table 10

**Supplementary Table 10.** Accession specific TE insertions.

| Query# | Reference# | No. of TE insertion | No. of TE affected genes |
|---|---|---|---|
| Wm82_v2 | W05 | 2,396 | 361 |
| W05 | Wm82_v2 | 3,009 | 419 |
| ZH13 | W05 | 2,465 | 350 |
| W05 | ZH13 | 2,335 | 400 |

# When the Query genome is compared to the Reference genome, TE insertion in the Query genome were identified.

## Supplementary Table 11

**Supplementary Table 11**. Summary of large structural variations (>100 Kb) between W05 and the other two genomes.

| Variation type | Wm82_v2 vs W05 | ZH13 vs W05 | Shared events |
|---|---|---|---|
| Inversion | 21 | 11 | 8 |
| Intra-chromosome translocation | 10 | 0 | 0 |
| Inter-chromosome translocation | 1 | 1 | 1 |
| Total | 32 | 12 | 9 |

## Supplementary Table 12

**Supplementary Table 12.** Primers used in this study.

| Target | Name | Orientation | Sequence (5' - 3') |
|---|---|---|---|
| Inversion junction in Wm82 | Junction | Forward | GGCCTGTTGTAGTGGAAAATTC |
| | | Reverse | CCTGGATACAGGTACGTTTTACT |
| Wm82 and W05 shared region at *I* locus | DNA input control | Forward | GCAAATGTTCATATCACTGAC |
| | | Reverse | CTTGTCAAGAGATCTAGTTCAC |
| Soybean *Actin11* | *GmACT11* | Forward | CGGTGGTTCTATCTTGGCATC |
| | | Reverse | GTCTTTCGCTTCAATAACCCTA |
| Subtilisin-anti-*CHS1* chimeric transcript | Subtilisin-anti-*CHS1* | Forward | GTTGTCAGTGTTCGAGAACC |
| | | Reverse | CGTTGAGACTGTTGTGCTCC |

# References


1. Kim MY*, et al.* Whole-genome sequencing and intensive analysis of the undomesticated soybean (*Glycine soja* Sieb. and Zucc.) genome. *Proc Natl Acad Sci U S A* **107**, 22032-22037 (2010).

2. Li YH*, et al. De novo* assembly of soybean wild relatives for pan-genome analysis of diversity and agronomic traits. *Nat Biotechnol* **32**, 1045-1052 (2014).

3. Qiu J*, et al.* Genome re-sequencing of semi-wild soybean reveals a complex *Soja* population structure and deep introgression. *Plos One* **9**, e108479 (2014).

4. Shimomura M*, et al.* The *Glycine max* cv. Enrei genome for improvement of Japanese soybean cultivars. *Int J Genomics* **2015**, 358127 (2015).

5. Schmutz J*, et al.* Genome sequence of the palaeopolyploid soybean. *Nature* **463**, 178-183 (2010).

6. Shen Y*, et al. De novo* assembly of a Chinese soybean genome. *Sci China Life Sci* **61**, 871-884 (2018).
