# Supplementary material for: A reference-grade wild soybean genome
Source: Nat Commun. 2019 Mar 14;10:1216. doi: 10.1038/s41467-019-09142-9 (PMC6418295; doi:10.1038/s41467-019-09142-9)
Supplement: Supplementary file 3 — Reporting Summary [file 41467_2019_9142_MOESM3_ESM.pdf]

nature research

Corresponding author(s): Hon-Ming Lam, Ting-Fung Chan

Reporting Summary

Nature Research wishes to improve the reproducibility of the work that we publish. This form provides structure for consistency and transparency in reporting. For further information on Nature Research policies, see Authors & Referees and the Editorial Policy Checklist.

Statistical parameters

When statistical analyses are reported, confirm that the following items are present in the relevant location (e.g. figure legend, table legend, main text, or Methods section).

n/a	Confirmed	
☐	☒	The exact sample size (*n*) for each experimental group/condition, given as a discrete number and unit of measurement
☐	☒	An indication of whether measurements were taken from distinct samples or whether the same sample was measured repeatedly
☒	☐	The statistical test(s) used AND whether they are one- or two-sided<br>*Only common tests should be described solely by name; describe more complex techniques in the Methods section.*
☒	☐	A description of all covariates tested
☒	☐	A description of any assumptions or corrections, such as tests of normality and adjustment for multiple comparisons
☒	☐	A full description of the statistics including central tendency (e.g. means) or other basic estimates (e.g. regression coefficient) AND variation (e.g. standard deviation) or associated estimates of uncertainty (e.g. confidence intervals)
☒	☐	For null hypothesis testing, the test statistic (e.g. *F*, *t*, *r*) with confidence intervals, effect sizes, degrees of freedom and *P* value noted<br>*Give P values as exact values whenever suitable.*
☒	☐	For Bayesian analysis, information on the choice of priors and Markov chain Monte Carlo settings
☒	☐	For hierarchical and complex designs, identification of the appropriate level for tests and full reporting of outcomes
☒	☐	Estimates of effect sizes (e.g. Cohen's *d*, Pearson's *r*), indicating how they were calculated
☒	☐	Clearly defined error bars<br>*State explicitly what error bars represent (e.g. SD, SE, CI)*

Our web collection on statistics for biologists may be useful.

Software and code

Policy information about availability of computer code

Data collection	SmartGrain (v1.1), Bionano AutoDetect software (v2.1.4)
Data analysis	MECAT (v1.0), SMRTLink (release 4.0.0.190159), BWA (v0.7.15-r1142), Picard (v2.9.0-1-gf5b9f50), Pilon (v1.22), HiRise (Dovetail Genomics LLC), Bionano Solve (v3.0.1 release v06082017), BLASR (v1.3.1.142244), PBSuite(v15.8.24), BLAST (v2.2.31), OMBlast (v1.4a), OMTools (v1.4a), BLAT (v35), BUSCO (v3.0.2), isPCR, e-PCR (v2.3.9), TRF (v4.0.4), RepeatMasker (v4.0.7), Repeatmodeler (v1.0.10), Exonerate (v2.4.0), trim_galore (v0.4.1), Trinity (v2.4.0), PASApipeline (v2.2.0), smrtanalysis (v2.3.0.140936), proovread (v2.13.12), Augustus (v3.2.3), Maker (v2.31.9), InterProScan (v5.29-68.0), tRNAScan-SE (v1.3.1), infernal (v1.1.2), Samtools (v1.2), R/qtl package (v1.41.6), QTL Cartographer (v1.17j), mummer (v4.0), BINGO (v3.0.3), Juicer (v1.5), Juicebox (v1.5.2)

For manuscripts utilizing custom algorithms or software that are central to the research but not yet described in published literature, software must be made available to editors/reviewers upon request. We strongly encourage code deposition in a community repository (e.g. GitHub). See the Nature Research guidelines for submitting code & software for further information.

April 2018

Data

Policy information about availability of data

All manuscripts must include a data availability statement. This statement should provide the following information, where applicable:

- Accession codes, unique identifiers, or web links for publicly available datasets
- A list of figures that have associated raw data
- A description of any restrictions on data availability

Data supporting the findings of this work are available within the paper and its Supplementary Information files. A reporting summary for this article is available as a Supplementary Information file. Genome assembly and annotations data of Glycine soja W05 were deposited in the DDBJ/ENA/GenBank under accession "QZWG00000000 [https://www.ncbi.nlm.nih.gov/assembly/GCA_004193775.1/]". The version described here is version QZWG01000000. All the raw sequencing reads were deposited in the NCBI Sequence Read Archive database under the accession “SRP158454 [https://www.ncbi.nlm.nih.gov/sra/?term=SRP158454]”. The optical molecules and optical contigs of W05 were deposited as NCBI Supplementary Files under accession “SUPPF_0000002760[ftp://ftp.ncbi.nlm.nih.gov/pub/supplementary_data/bionanomaps.csv]” and “SUPPF_0000002761[ftp://ftp.ncbi.nlm.nih.gov/pub/supplementary_data/bionanomaps.csv]”. The optical contigs of other soybean accessions were deposited as NCBI Supplementary Files under accessions “SUPPF_0000002797-SUPPF_0000002807[ftp://ftp.ncbi.nlm.nih.gov/pub/supplementary_data/bionanomaps.csv]”. W05 genome assembly and annotation are also available at wildsoydb database (www.wildsoydb.org/Gsoja_W05). Request for seeds of the parental line C08 and the RI lines reported in this manuscript could be sent to Hon-Ming Lam (honming@cuhk.edu.hk). Seeds of W05 could not be freely distributed to outside China due to a legal restriction in the exchange of wild germplasms. The datasets generated and analyzed during the current study are available from the corresponding author on reasonable request. The source data for Figure 2b and Supplementary Figure 6 are provided as a Source Data file.

Field-specific reporting

Please select the best fit for your research. If you are not sure, read the appropriate sections before making your selection.

☒ Life sciences ☐ Behavioural & social sciences ☐ Ecological, evolutionary & environmental sciences

For a reference copy of the document with all sections, see nature.com/authors/policies/ReportingSummary-flat.pdf

Life sciences study design

All studies must disclose on these points even when the disclosure is negative.

Sample size	For genome assembly, annotation, evaluation, and comparison, no sample size predetermination is needed.<br>For QTL mapping, no sample size predetermination is needed in this study.
Data exclusions	No data was excluded.
Replication	For genome assembly, annotation, evaluation, and comparison, no replication is needed.<br>For QTL mapping, biological replicates of each trait from at least two years were used.
Randomization	For genome assembly, annotation, evaluation, and comparison, no randomization is needed.<br>For QTL mapping, a core panel of 96 RI lines that exhibited a diverse spectrum of growth period duration was selected for sequencing and mapping analyses (Qi et al Nat Comm 2014).
Blinding	There is no blinding. Sequencing, optical mapping, and phenotype data were collected/analyzed in systematic way which involved no bias.

Reporting for specific materials, systems and methods

Materials & experimental systems

n/a	Involved in the study	
☐	☒	Unique biological materials
☒	☐	Antibodies
☒	☐	Eukaryotic cell lines
☒	☐	Palaeontology
☒	☐	Animals and other organisms
☒	☐	Human research participants

Methods

n/a	Involved in the study	
☒	☐	ChIP-seq
☒	☐	Flow cytometry
☒	☐	MRI-based neuroimaging

Unique biological materials

Policy information about availability of materials

Obtaining unique materials	Request for seeds of the parental line C08 and the RI lines reported in this manuscript could be sent to Hon-Ming Lam (honming@cuhk.edu.hk). Seeds of W05 could not be freely distributed to outside China due to a legal restriction in the exchange of wild germplasms.
