# Supplementary material for: A reference-grade wild soybean genome
Source: Nat Commun. 2019 Mar 14;10:1216. doi: 10.1038/s41467-019-09142-9 (PMC6418295; doi:10.1038/s41467-019-09142-9)
Supplement: Supplementary file 4 — Description of Additional Supplementary Data [file 41467_2019_9142_MOESM4_ESM.docx]

**Description of Additional Supplementary Files:**

File Name: Supplementary Data 1
Description: Position of SSR markers in W05 and Wm82_v2 genomes.

File Name: Supplementary Data 2
Description: Summary of RNA-seq and PacBio IsoSeq data. For RNA-seq data downloaded from NCBI SRA database, SRR accession codes could be found in the Note column.

File Name: Supplementary Data 3
Description: Major QTL identified with W05 or Wm82_v2 genome as reference.

File Name: Supplementary Data 4
Description: Comparison of sequence of selected genes in W05 and Wm82_v2.

File Name: Supplementary Data 5
Description: GO enrichment of accession specific TE affected genes.

File Name: Supplementary Data 6
Description: Sample information, data generation and de novo OM assembly for multiple soybean accessions

**Description of Source Data Files:**

Excel Sheet Name: Figure 2b

Description: Unprocessed gel image for Figure 2b. NTC: no template control.

Excel Sheet Name: Supplementary Figure 6

Description: Unprocessed gel image for Supplementary Figure 6.
