# Supplementary material for: A reference-grade wild soybean genome
Source: Nat Commun. 2019 Mar 14;10:1216. doi: 10.1038/s41467-019-09142-9 (PMC6418295; doi:10.1038/s41467-019-09142-9)
Supplement: Supplementary file 2 — Peer Review [file 41467_2019_9142_MOESM2_ESM.pdf]

Editorial Note: this manuscript has been previously reviewed at another journal that is not operating a transparent peer review scheme. This document only contains reviewer comments and rebuttal letters for versions considered at *Nature Communications.*

REVIEWERS' COMMENTS:

Reviewer #1 unavailable.

Reviewer #2 (Remarks to the Author):

The manuscript provides a relative high quality soybean genome. But comparing with previous soybean genomes and reported related studies, most of the results from this the manuscript have been referenced and are expected. They are lack of novelty.

Reviewer #3 (Remarks to the Author):

Very poorly written.

What about 'Lee' and others coming?

In the Abstract you violated 8 of my 26 Golden Rules. See below for a list, reasons my own manuscripts were rejected. Fix those and I can look at the data. You do not want me to be irritated, it goes badly.

1. All Paragraphs in the Introduction and Discussion need a reference after the first sentence and whenever the source is changed.
2. References should largely match between the Introduction and Discussion sections.
3. All enzymes need a EC number at first mention
4. All manufactures have (City, State) after first mention.
5. Don't start sentences with indefinite articles like "this, It, These.
6. Remove all uses of the possessive voice "we, our etc."
7. Do start sentence with The… on occasion
8. Don't start sentences with numbers
9. Replace levels with a non-nautical word, amount or concentration or layer
10. Every % has a unit directly afterward (v/v) etc. and a descriptor before.
11. Replace all –ings with –eds will help tense consistency
12. Define all abbreviations at first use
13. Varieties are in 'parentheses' at first mention.
14. Do not use gene expression, or up/down regulated; be specific use increased or decreased abundance, initiation elongation, activity or whatever the measure was.
15. Match plurals within sentences.
16. Describe the source of materials and conditions of growth.
17. Describe the number of replicates, replications and repeats
18. Use shown do not use revealed, nothing was deliberately hiddden.
19. Use ; instead of abutted)(s
20. Capitals within sentences are for Names only
21. Separate units from numbers ie. 3 % not 3% (OK in some Journals), 4 mg not 4mg etc.
22. Use many references from the Introduction in the Discussion.
23. Commas separate numbers over 999
24. Italics for all species names, with attributions
25. Centrifuge units are italic g not rpm
26. DNA is kbp RNA is kb

[Editor: Reviewer #3 wrote in Remark to Editor section that the analysis conducted in this study, including those concerned by previous Reviewer #1, was good.]

REVIEWERS' COMMENTS:

Reviewer #1 unavailable.

Reviewer #2 (Remarks to the Author):

The manuscript provides a relative high quality soybean genome. But comparing with previous soybean genomes and reported related studies, most of the results from this the manuscript have been referenced and are expected. They are lack of novelty.

Response:
The main contribution of this manuscript is to provide a high-quality reference-grade wild soybean genome that can be used by legume scientists for comparative genomic analysis and soybean breeders for crop improvement. There are several examples reported in this manuscript to exemplify the quality and potential applications of this genome resource.

Reviewer #3 (Remarks to the Author):

Very poorly written.

Response:
A native English-speaking author has performed another round of proofreading for this manuscript to correct typos and other mistakes.

What about 'Lee' and others coming?

Response:
We have revised Supplementary Table 3 to include assembly information of Lee and other publicly available soybean assemblies for comparison. Our wild soybean genome W05 and the cultivated soybean genome ZH13 have the longest contig N50 among all assembled genomes.

In the Abstract you violated 8 of my 26 Golden Rules. See below for a list, reasons my own manuscripts were rejected. Fix those and I can look at the data. You do not want me to be irritated, it goes badly.

1. All Paragraphs in the Introduction and Discussion need a reference after the first sentence and whenever the source is changed.
2. References should largely match between the Introduction and Discussion sections.
3. All enzymes need a EC number at first mention
4. All manufactures have (City, State) after first mention.
5. Don't start sentences with indefinite articles like "this, It, These.
6. Remove all uses of the possessive voice "we, our etc."
7. Do start sentence with The… on occasion
8. Don't start sentences with numbers

9. Replace levels with a non-nautical word, amount or concentration or layer
10. Every % has a unit directly afterward (v/v) etc. and a descriptor before.
11. Replace all –ings with –eds will help tense consistency
12. Define all abbreviations at first use
13. Varieties are in ‘parentheses’ at first mention.
14. Do not use gene expression, or up/down regulated; be specific use increased or decreased abundance, initiation elongation, activity or whatever the measure was.
15. Match plurals within sentences.
16. Describe the source of materials and conditions of growth.
17. Describe the number of replicates, replications and repeats
18. Use shown do not use revealed, nothing was deliberately hiddden.
19. Use ; instead of abutted)(s
20. Capitals within sentences are for Names only
21. Separate units from numbers ie. 3 % not 3% (OK in some Journals), 4 mg not 4mg etc.
22. Use many references from the Introduction in the Discussion.
23. Commas separate numbers over 999
24. Italics for all species names, with attributions
25. Centrifuge units are italic g not rpm
26. DNA is kbp RNA is kb

Response:
We have revised our Abstract and performed another round of proofreading for this manuscript.
